# CENP-V is required for proper chromosome segregation through interaction with spindle microtubules in mouse oocytes

**DOI:** 10.1038/s41467-021-26826-3

**Published:** 2021-11-11

**Authors:** Dalileh Nabi, Hauke Drechsler, Johannes Pschirer, Franz Korn, Nadine Schuler, Stefan Diez, Rolf Jessberger, Mariola Chacón

**Affiliations:** 1 Institute of Physiological Chemistry, Medical Faculty Carl Gustav Carus, Technische Universität Dresden, Dresden, Germany; 2 B CUBE-Center for Molecular Bioengineering, Technische Universität Dresden, Dresden, Germany; 3 Cluster of Excellence Physics of Life, Technische Universität Dresden, Dresden, Germany; 4 Max Planck Institute of Molecular Cell Biology and Genetics, Dresden, Germany; 5 Present Address: Department of Neuropediatrics Charité-Universitätsmedizin Berlin, Freie Universität Berlin, Humboldt-Universität zu Berlin, and Berlin Institute of Health, Berlin, Germany; 6 Present Address: CABIMER, Centro Andaluz de Biología Molecular & Medicina Regenerativa, Sevilla, Spain

**Keywords:** Genomic instability, Checkpoints, Meiosis, Kinetochores, Oogenesis

## Abstract

Proper chromosome segregation is essential to avoid aneuploidy, yet this process fails with increasing age in mammalian oocytes. Here we report a role for the scarcely described protein CENP-V in oocyte spindle formation and chromosome segregation. We show that depending on the oocyte maturation state, CENP-V localizes to centromeres, to microtubule organizing centers, and to spindle microtubules. We find that *Cenp-V*^*−/−*^ oocytes feature severe deficiencies, including metaphase I arrest, strongly reduced polar body extrusion, increased numbers of mis-aligned chromosomes and aneuploidy, multipolar spindles, unfocused spindle poles and loss of kinetochore spindle fibres. We also show that CENP-V protein binds, diffuses along, and bundles microtubules in vitro. The spindle assembly checkpoint arrests about half of metaphase I *Cenp-V*^*−/−*^ oocytes from young adults only. This finding suggests checkpoint weakening in ageing oocytes, which mature despite carrying mis-aligned chromosomes. Thus, CENP-V is a microtubule bundling protein crucial to faithful oocyte meiosis, and *Cenp-V*^*−/−*^ oocytes reveal age-dependent weakening of the spindle assembly checkpoint.

## Abstract (web summary)

Chromosome segregation is essential to avoid aneuploidy, yet in mammalian oocytes it progressively fails in an age-dependent manner. Here the authors identify CENP-V as a microtubule binding and bundling protein crucial to faithful oocyte meiosis, and present Cenp-V−/− oocytes as revealing age-dependent weakening of the spindle assembly checkpoint.

## Introduction

Mammalian oogenesis is burdened with comparably high rates of failures that may result in chromosome mis-segregation and aneuploidy (for reviews see^1–9^). These errors happen during oocyte meiosis, which includes a very prolonged period of arrest, and increase with advancing age. Several mechanisms were proposed to contribute to this problem. One prominent mechanism is a gradual deterioration of cohesin (for reviews see^6,7,10–12^), which ought to maintain sister chromatid cohesion throughout oocyte lifespan. Cohesin is loaded onto oocyte chromosomes at the entry into meiosis with no or very little replenishment later. The increasing loss of sister chromatid cohesion with consequently loss of chiasmata in meiosis I and weakening of sister centromere cohesion up to anaphase II is considered a major factor in age-dependent oocyte chromosome mis-segregation. Several other mechanisms likely also contributes to oocyte ageing and problems associated with it (for reviews see^7,13–19^). Telomere damage also increases with progressing age and may cause chromosome end fusions and thus aberrant chromosome segregation. Likewise, erroneous attachment of microtubules to the kinetochores may lead to mis-segregation if not corrected during a spindle assembly checkpoint (SAC) mediated arrest. It appears as if the control mechanisms provided by the SAC also weaken with advancing age although this has not yet been clearly demonstrated. Thus, for persistent maintenance of oocyte chromosome integrity and proper segregation a well-balanced system of monitoring, error correction, and strict control pathways are required. This, however, is not fully sufficient as it fails in the long term. In this communication we describe an additional factor that plays a central role in the control of oocyte meiosis, CENP-V.

Human CENP-V is a 275 amino acid protein described so far in only very few publications, initially by William Earnshaw in 2008. This group identified it as a kinetochore component in proteome analysis of human mitotic chromosomes^20^. The 29.7 kDa protein CENP-V (pI 9.8) is highly conserved among vertebrates. In HeLa cells, it localizes to the mitotic kinetochores from prometaphase to metaphase and moves to the spindle mid-zone in anaphase. Cysteines within CENP-V may coordinate Zn^++^ which possibly mediates DNA binding. When overexpressed in HeLa cells, CENP-V causes hypercondensation of pericentromeric heterochromatin. Knock-down of CENP-V in HeLa cells leads to decompaction of chromosomes, decondensation of the heterochromatin. Metaphase alignment of chromosomes in CENP-V depleted HeLa cells is impaired and lagging chromosomes appear in anaphase^20^. Out of the context of mitosis, CENP-V appears to be involved in the regulation of microtubule structures as it promotes cell migration by targeting Src family kinases to the microtubule network in the leading edge of migrating cells^21^. CENP-V also supports tubulin acetylation at the basal bodies and axonemes of primary cilia^22^ – a posttranslational tubulin modification that is thought to enhance the resilience of microtubules to mechanical stress^23^.

In this work, we investigated the role of CENP-V in oocytes as their particularly delicate meiotic and spindle features may require functional CENP-V during maturation. We describe CENP-V as an important regulator of oocyte microtubule-kinetochore associations and chromosome segregation, whose absence causes age-dependent failure of the SAC and aneuploidy.

## Results

### CENP-V changes its localization during meiosis in mouse oocytes

**Fig. 1:**
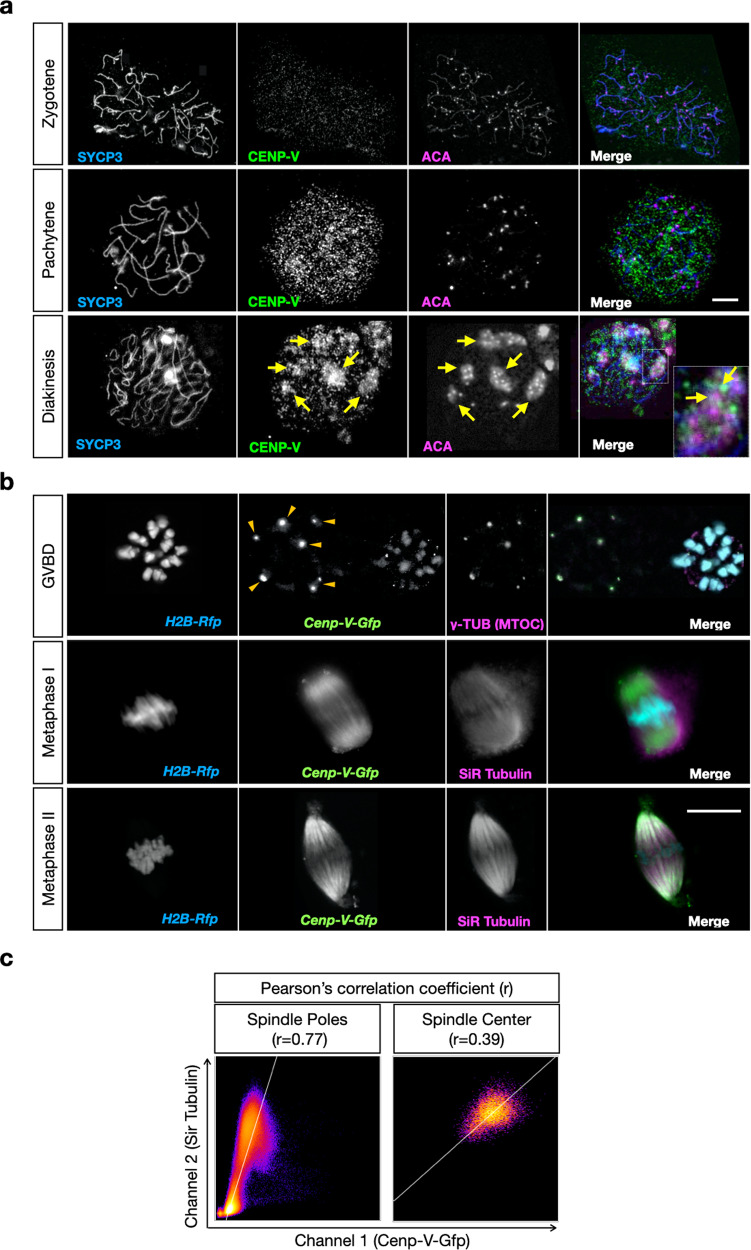
CENP-V localises to the centromere regions during meiotic prophase and to MTOCs, chromosomes, and spindle during oocyte maturation. **a** CENP-V distribution during meiotic prophase in *Cenp-V*^*+/+*^ embryonic chromosome spreads by immunofluorescent staining. Chromosome axes (AEs/LEs) were stained with anti-SYCP3 (blue); CENP-V was probed with anti-CENP-V (green); centromeres and surrounded regions by anti-ACA. CENP-V signal started to appear at pachytene and concentrates at the centromere regions (yellow arrows) at diakinesis; *n* = 19 cells from 2 independent experiments. Scale bar = 5 μm. **b** CENP-V distribution during mouse oocyte maturation in whole cells. Live imaging performed with *Cenp-V*^*+/+*^ oocytes injected by mRNA of *H2B-Rfp* (blue) and *Cenp-V-Gfp* (green). Maximum projections from representative oocytes at germinal vesicle break down (GVBD), metaphase I, and metaphase II stages are shown. Scale bar = 10 μm. Some GVBD cells were fixed and immunostained with anti γ-Tubulin as an MTOC marker. Spindle at metaphase I and II were visualised by SiR Tubulin dye. Polar body is indicated as PB. Note that at the early stages of maturation CENP-V distributes along the entire chromosomes and colocalizes with MTOCs (yellow arrow heads). At metaphase I and II CENP-V appears mainly in the spindle and enriches at the spindle poles. See also supplementary movie 1; *n* = 12 cells from 3 independent experiments. **c** Quantification of colocalization of CENP-V with the spindle pole by determining the respective Pearson’s correlation coefficient; *n* = 10 cells from 3 independent experiments; the color code highlights the frequency of dots present in a certain region of the scatter plot (from blue to yellow and white with increasing frequencies).

CENP-V has been described as a component of the kinetochore from prometaphase to metaphase during mitosis^20^. To test whether this localisation is conserved in meiotic prophase I we stained mouse embryonic oocyte spreads with an anti-CENP-V antibody (Fig. 1a) raised against the full-length mouse protein and validated on CENP-V deficient cells (see next section). Unless otherwise specified, all experiments described below were done using young adults, i.e. 6 weeks to 3 months old mice. In wild-type (wt) mice we observed CENP-V to appear in pachytene yielding a diffuse signal. At the end of meiotic prophase in diakinesis CENP-V was found in the vicinity of centromeres and the surrounding heterochromatin as observed by co-staining with the centromere marker ACA (Fig. 1a). This pattern is similar to that seen in spermatocyte prophase I, where CENP-V localized to the centromere region and external to the centromere protein CENP-A from the pachytene stage onwards, similar to what has been described for mitotic HeLa cells^20^ (Sup. Figure 1). However, CENP-V was excluded from the spermatocyte X/Y chromosomes, the sex body chromatin.

**Fig. 2:**
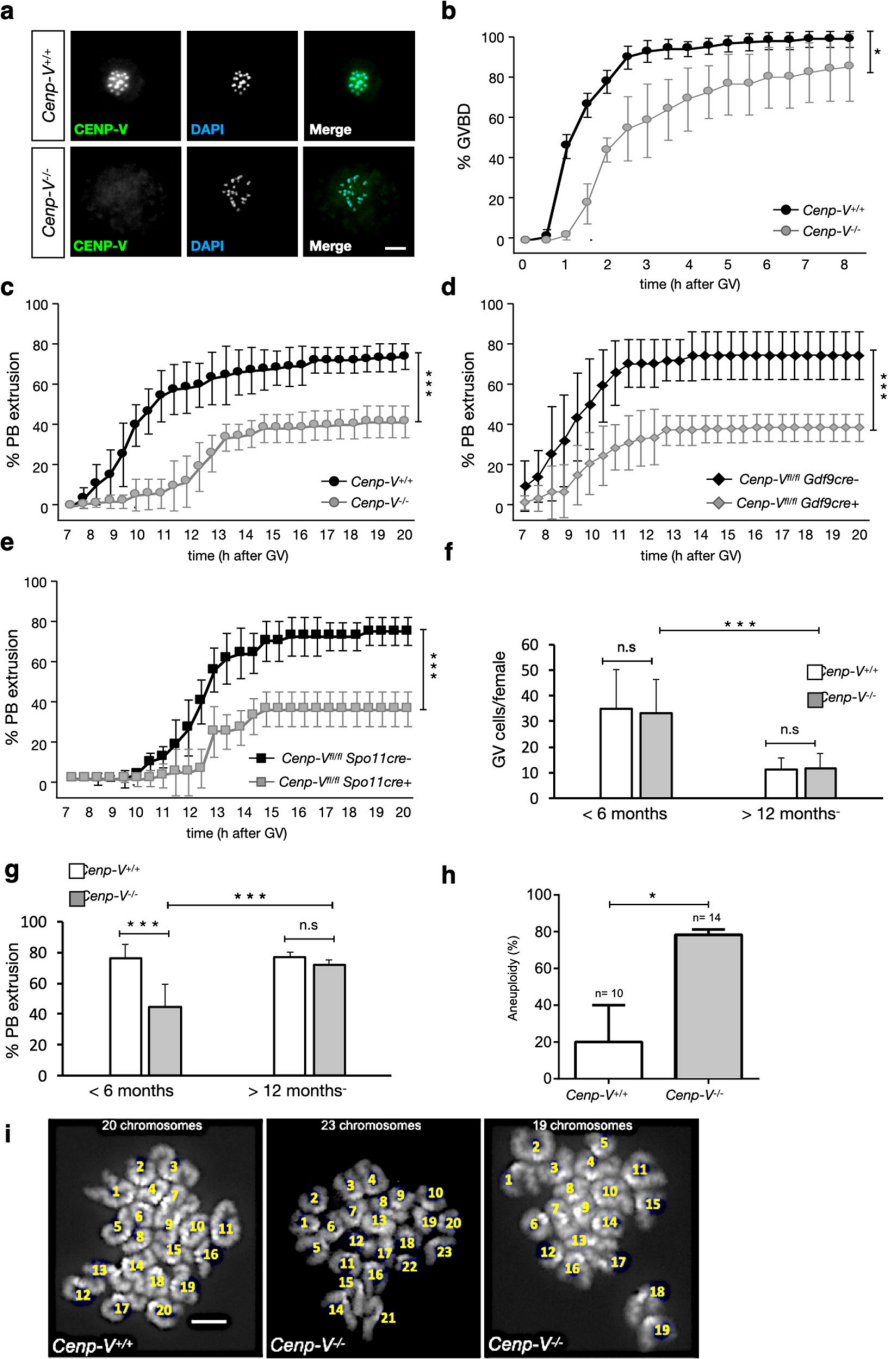
Cenp-V−/− oocytes are impaired in MI progression. **a** Expression of CENP-V protein in *Cenp-V*^*+*/+^ and *Cenp-V*^*−/−*^ oocytes. CENP-V is probed by anti CENP-V antibody (green), DNA is stained by DAPI (blue).Scale bar = 5 μm. **b** Cumulative percentage of GVBD cells over time in *Cenp-V*^*+/+*^ and *Cenp-V*^*−/−*^ oocytes. **c** Cumulative percentage of first polar body (PB) extrusion over time in *Cenp-V*^*+/+*^ *and Cenp-V*^*−/−*^ oocytes. **d** Cumulative percentage of first polar body extrusion over time in *Cenp-V*^*fl/fl*^ *Gdf9cre-* and *Cenp-V*^*fl/fl*^ *Gdf9cre* + oocytes. **e** Cumulative percentage of first polar body extrusion over time in *Cenp-V*^*fl/fl*^ *Spo11cre-* and *Cenp-V*^*fl/fl*^ *Spo11cre* + oocytes. **f** Number of oocytes in young (< 6 months) and old (>12 months) *Cenp-V*^*+/+*^ and *Cenp-V*^*−/−*^ females as an average number of GV cells per female, and corresponding SD. **g** Percentage of first polar body (PB) extrusion after 20 h culture of oocytes from young (< 6 months) and old (>12 months) *Cenp-V*^*+/+*^ and *Cenp-V*^*−/−*^ females, shown as an average percentage per female. **h** Percentage of MII oocytes showing aneuploidy. **i** Examples of MII oocyte chromosome spreads of control and *Cenp-V*^*−/−*^ oocytes. The numbers of chromosomes are indicated; size bar = 50 μm. Statistic: (**b**–**h**); data were expressed as mean ± SD and statistical differences were tested by a paired two tailed *T*-test (***p < 0.001, **p < 0.01, *p < 0.05). The exact *p* values are available in the source data file.*T-test* results between *Cenp-V*^*−/−*^ (c), *Cenp-V*^*fl/fl*^ *Gdf9cre* (**d**) *Cenp-V*^*fl/fl*^ *Spo11cre (e)* lines were not significant, i.e. they all showed the same phenotype. (b); statistical differences refer to the average of the data points between 1 and 5 h after GV. (**c**,**d**,**e**,**f**); statistical differences refer to the average of all data points. (**b**, **c**, **f**, **g**) *n* = 303 oocytes from 6 independent experiments; (d,**e**) *n* = 134 oocytes from 3 independent experiments and (**i**) *n* = 20 cells from 3 independent experiments.

The distribution of CENP-V during meiotic resumption was analyzed in mouse oocytes at different stages of maturation. To allow fixed and live-cell imaging, oocytes expressing CENP-V-GFP and H2B-RFP after injection of *Cenp-V-Gfp* and *H2B-Rfp* mRNA at the arrested germinal vesicle (GV) stage were used (Fig. 1b). At the stage of germinal vesicle break down (GVBD) the CENP-V-GFP signal covered the entire chromosomes. Since Honda et al. suggested an association of CENP-V with microtubules^21^, some GVBD oocytes were fixed and probed with an anti γ-tubulin antibody as a marker for microtubule organising centres (MTOCs). Metaphase I and II spindles were stained with SiR Tubulin (Fig. 1b and supplementary mov. S1). During meiotic resumption we observed three different pools of CENP-V: (i) at the chromosomes, (ii) at the MTOCs (Fig. 1b, upper row), and (iii) at the meiotic spindles. At metaphase I CENP-V is mainly enriched at the spindle and this was maintained throughout metaphase II (Fig. 1b, middle and lower rows). To get an unbiased quantification of the localization of CENP-V to the spindle, we measured the Pearson’s coefficient, using an imaging algorithm to measure the colocalization along z-stacks between CENP-V-GFP and SiR Tubulin. Notably, in the spindle pole Pearson’s coefficient was close to 1, indicating an almost complete colocalization. On the contrary, colocalization of CENP-V and SiR Tubulin decreased towards the spindle center, suggesting that CENP-V does not show a homogenous distribution in the spindle but rather accumulates at the pole (Fig. 1c). The same pattern was observed by staining fixed oocytes with an anti-CENP-V antibody (Fig. 2a, Sup. Figure 2).

This suggests that during female meiosis CENP-V expands its localization from around the centromere region in prophase to along metaphase I chromosomes, to the MTOCs, and to microtubules during metaphase I and PB extrusion (PBE).

### Deletion of *Cenp-V* arrests mouse oocytes at metaphase I

Would the lack of CENP-V affect oocytes maturation, meiotic progression? CENP-V homozygous null mice (*Cenp-V*^*−/−*^*)* were generated by *Cre* recombinase-mediated deletion of the floxed exon 3 of *Cenp-V*, confirmed by DNA sequencing. We first assessed the loss of CENP-V in *Cenp-V*^*−/−*^ mice by immunofluorescence (IF) and western blot (WB) using commercial and in-house generated anti-CENP-V antibodies. The absence of CENP-V signals from chromosome spreads and from WB of *Cenp-V*^*−/−*^ oocytes or ovary confirmed loss of the protein, and these experiments also validated the antibodies (Fig. 2a, Sup. Figure 3a, b). *Cenp-V*^*−/−*^ mice were viable and females but not males were subfertile. Subfertility was observed when *Cenp-V*^*−/−*^ females were bred with wt, heterozygous, or homozygous CENP-V deficient males (Sup. Figure 4a). Because we observed subfertility only in *Cenp-V*^*−/−*^ females we focused on the role of CENP-V in oocyte meiosis.

*Cenp-V*^*+/+*^ *and Cenp-V*^*−/−*^ oocytes were collected, maintained, and synchronized in prophase with intact germinal vesicles (GVs) by incubation in milrinone-containing medium. Two hours after removal of milrinone *Cenp-V*^*+/+*^ *and Cenp-V*^*−/−*^ oocytes underwent germinal vesicle breakdown (GVBD), i.e. meiotic resumption. In average, *Cenp-V*^*−/−*^ oocytes required significantly longer to undergo germinal vesicle breakdown (GVBD) (Fig. 2b; *Cenp-V*^*+/+*^: 83.7% ± 16.18 at 2.5 h; *Cenp-V*^*−/−*^: 54% ± 15.33 at 2.5 h). Twenty hours after the GV stage, 74.3% ± 6.24 of *Cenp-V*^*+/+*^ but only 41.6% ± 7.95 of *Cenp-V*^*−/−*^ oocytes have extruded the first polar body (PB). This process was also significantly slower in *Cenp-V*^*−/−*^ oocytes than in *Cenp-V*^*+/+*^ oocytes (Fig. 2c). To test whether this reduction in PB extrusion was due to a specific role of CENP-V during meiosis and not due to an unspecific effect brought about by the absence of CENP-V from all tissues in the *Cenp-V*^*−/−*^ mouse strain we performed similar experiments using a *Cenp-V*^*fl/fl*^ conditional deletion strain crossed with GDF9-iCre or SPO11-Cre. The oocyte-specific GDF9-iCre expresses Cre shortly after birth, i.e. in oocytes that have just reached dictyate arrest^24^. Spo11-Cre is expressed shortly after entry into meiosis^25^. The decrease in PBE in *Cenp-V*^*fl/fl*^*,Gdf9-iCre* + and *Cenp-V*^*fl/fl*^*,Spo11-Cre* + mice was very similar to that seen in *Cenp-V*^*−/−*^ oocytes confirming the specific role of CENP-V during oocyte meiosis (Fig. 2d, e). Similar results were also obtained using Vasa-Cre^26^ which is expressed in oocytes at around early zygotene (not shown). Together this data suggests that CENP-V is not required in prophase I but is required after dictyate arrest.

To further check possible deleterious effects of CENP-V deficiency on oogenesis we quantified the number of GV cells per female and performed histological analysis of ovaries from *Cenp-V*^*+/+*^ and *Cenp-V*^*−/−*^ mice. No differences were observed in GV cell numbers, and follicular development appeared to be normal as evaluated by hematoxylin/eosin staining (Fig. 2f, left two columns, Sup. Figure 3b). To assess a possible role of CENP-V during ageing we quantified the GV cell numbers and performed life imaging experiments in *Cenp-V*^*−/−*^ females older than 12 months. We found a reduction of the GV cell number due to ageing but not due to the absence of CENP-V (Fig. 2f). Surprisingly, the reduction in PBE found in young *Cenp-V*^*−/−*^ oocytes vanished in oocytes from old females (Fig. 2g). With the control steady at app. 78 %, 44,7 ± 14,62 % of the young the *Cenp-V*^*−/−*^ *oocytes* underwent PBE compared to 71,7 ± 6,2 of old *Cenp-V*^*−/−*^ oocytes. Assuming that the errors caused by the absence of CENP-V remain the same in young and old oocytes, this hints at the weakening of the key control mechanism, the spindle assembly checkpoint (SAC). Together, these results suggest that CENP-V plays an important role for faithful oocyte maturation and chromosome segregation. Indeed, we observed an increased frequency of MII oocytes harboring abnormal numbers of pairs of sister chromatids, i.e. aneuploidy (Fig. 2h, i). To assess aneuploidy in meiosis II oocytes we counted chromosomes, i.e. pairs of sister chromatids, on MII metaphase chromosome spreads. Mis-segregation leads to loss of chromosomes or increased numbers and thus any deviation from 20 pairs of sister chromatids is considered an aneuploidy. This analysis revealed almost 4-fold increased rates of aneuploidy in absence of CENP-V. Sister chromatid cohesin itself was not lost since we did not detect single, unpaired chromatids. The limited number of oocytes present in aged mice precluded an extensive analysis of aneuploidy in those, but when mice of 9, 16, and 21 months of age were analyzed (together 15 oocytes for littermate controls and 10 oocytes for *Cenp-V*^*−/−*^ oocytes), we observed 46 % of cells for the control and 60 % of the *Cenp-V*^*−/−*^ oocytes showing aneuploidy. Generally, aneuploidy increases with age, for example through cohesion decay. While due to low numbers the increase in aged *Cenp-V*^*−/−*^ oocytes is only a trend, the data indicates a higher frequency of aneuploidy in aged CENP-V deficient oocytes which, however, contrary to young oocytes still proceed to the PB stage, consistent with the above data.

### Chromosome alignment is erroneous and delayed in *Cenp-V*^*−/−*^ oocytes

A failure to perform PBE could be triggered by chromosome mis-alignment which can lead to SAC activation and thus to arrest of oocytes before PBE^27^. Since CENP-V accumulated at the centromeric region and at the meiotic spindle, both key structures for proper chromosome alignment and kinetochore capture^28^, we decided to monitor chromosome alignment in the absence of CENP-V.

**Fig. 3:**
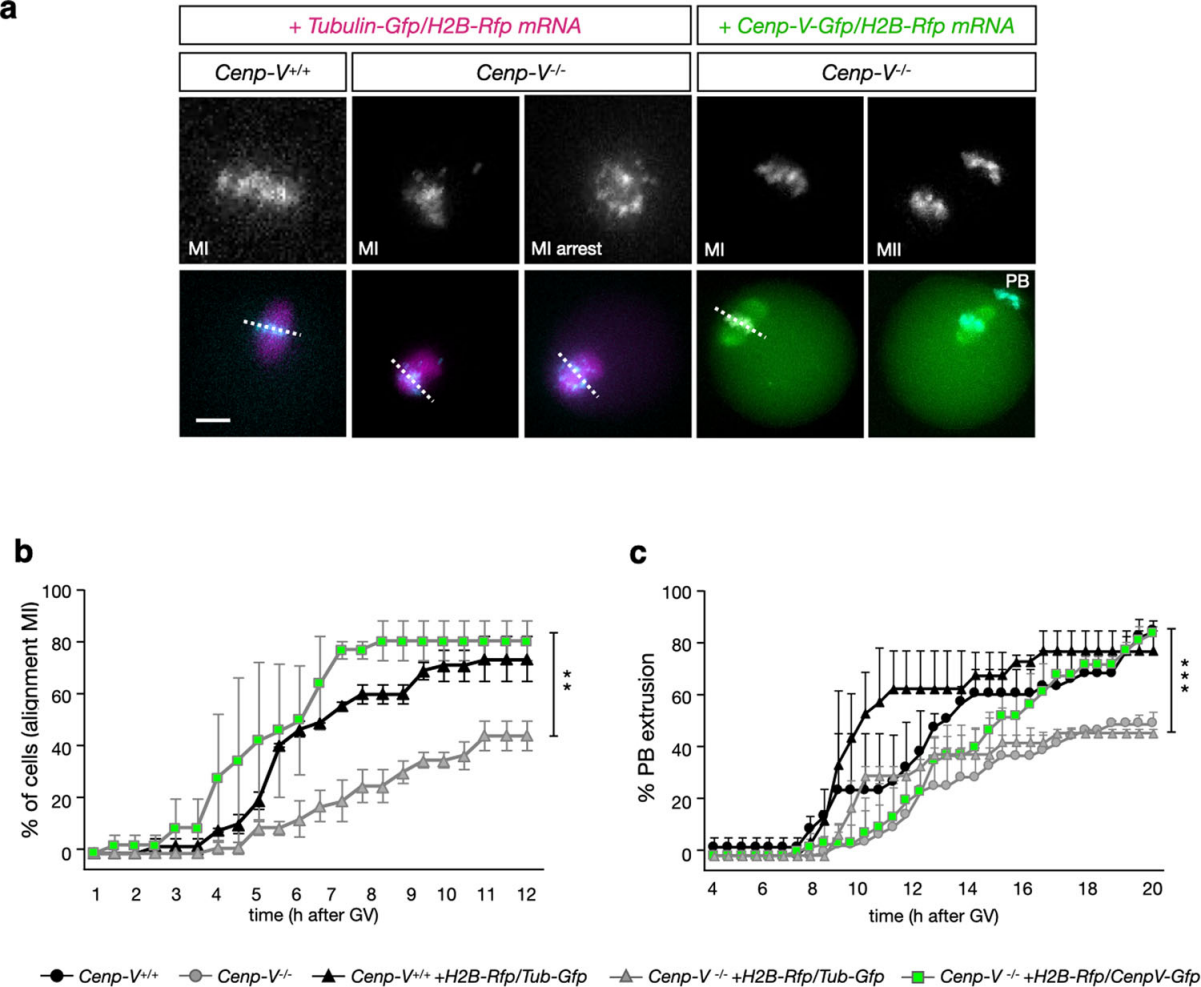
Chromosome alignment is reduced and delayed in Cenp-V−/− oocytes. Polar body extrusion and chromosome alignment return to control values upon Cenp-V-Gfp mRNA injection. (**a**) Selected time frames from a time-lapse experiment showing progression through meiotic prophase until metaphase II in *Cenp-V*^*+/+*^ and *Cenp-V*^*−/−*^ oocytes expressing β-TUBULIN-GFP (magenta), H2B-RFP (cyan, large image in grey) and/or CENP-V-GFP (green) after mRNA injection. At 09:30 h in metaphase I, the spindle is assembled and chromosomes are aligned in the equatorial plate (white dash line) in *Cenp-V*^*+/+*^ compare to *Cenp-V*^*−/−*^ oocyte. Note that *Cenp-V*^*−/−*^ oocytes show normal chromosome alignment and extrude the PB only when expressing CENP-V-GFP. Oocytes were imaged after 4 h release from GV every 30 m. Maximum intensity projection is shown. See also supplementary Movies S2,S3,S4). Scale bar = 10 µm. **b** Percentage of cells that show chromosome alignment overtime of the experiment shown in (**a**). in *Cenp-V*^*+/+*^ *and Cenp-V*^*−/−*^ oocytes. Quantification finishes before the first polar body extrusion. Note that at 10 h, 75 % of control oocytes have reached full chromosome alignment compared to the 35% of *Cenp-V*^*−/−*^ oocytes, and only the injection of *Cenp-V-Gfp* (green square line) mRNA restores the values to the control. **c** Cumulative percentage of first polar body extrusion overtime of the experiment shown in (a). Note that *Cenp-V*^*−/−*^ oocytes properly extrude the PB only when expressing CENP-V-GFP (green square line) and not when expressing β-TUBULIN-GFP/H2B-RFP. See also supplementary movie S1. Statistic: (**b**, **c**) data were expressed as mean ± SD and statistical differences were tested by a two way Anova test (***p < 0.001, **p < 0.01, *p < 0.05). The exact *p* values are available in the source data file. Statistical differences refer to the average of all data points. *Anova test* between *Cenp-V*^*+/+*^ and *Cenp-V*^*+/+*^ injected was not significant. *Anova test* between *Cenp-V*^*−/−*^ and *Cenp-V*^*−/−*^ *β-Tubulin-Gfp/H2B-Rfp* injected was not significant. *n* = 249 cells from 5 different experiments.

Oocytes proficient and deficient for CENP-V were analysed by time-lapse fluorescence imaging of microtubules (β-TUBULIN-GFP) and chromosomes (H2B-RFP) after injection of the respective mRNAs (Fig. 3 and supplementary mov. S2 and S3). The H2B-RFP signal was followed until the spindle reached the cortex prior to PBE (Fig. 3a). Ten hours after GV, 75% of the control cells showed full chromosome alignment at the metaphase plate compared to 40% of *Cenp-V*^*−/−*^ cells (Fig. 3b). These values were very similar to the respective frequencies of PBE (Fig. 3c) and to the values of PBE in non-injected cells (Figs. 2, 3c), suggesting that chromosome mis-alignment contributes to the decrease in PBE and that injection per se had no effect. To test whether the lack of CENP-V and no other cause reduced PBE and chromosome mis-alignment we also injected *Cenp-V-Gfp* mRNA into *Cenp-V*^*−/−*^ cells. Representative frames of the time course are shown in Fig. 3a, which includes an example of a *Cenp-V*^*−/−*^ oocyte overexpressing β-TUBULIN-GFP/H2B-RFP that stayed arrested at MI and a *Cenp-V*^*−/−*^ oocyte overexpressing CENP-V-GFP that successfully extruded its PB (supplementary mov. S3 compare to supplementary mov S4). *Cenp-V*^*−/−*^ oocytes expressing CENP-V-GFP restored both, chromosome alignment and PBE to control values (Fig. 3b, c), confirming that the lack of CENP-V causes the chromosome mis-alignment that leads to the reduction of PBE. For clarity of the results we do not represent the percentage of PBE and chromosome alignment of the *Cenp-V*^*+/+*^ oocytes expressing CENP-V-GFP as they behave like control oocytes (Fig. 1b, supplementary mov. S1).

### Inhibition of Mps1 restores progression to metaphase II

**Fig. 4:**
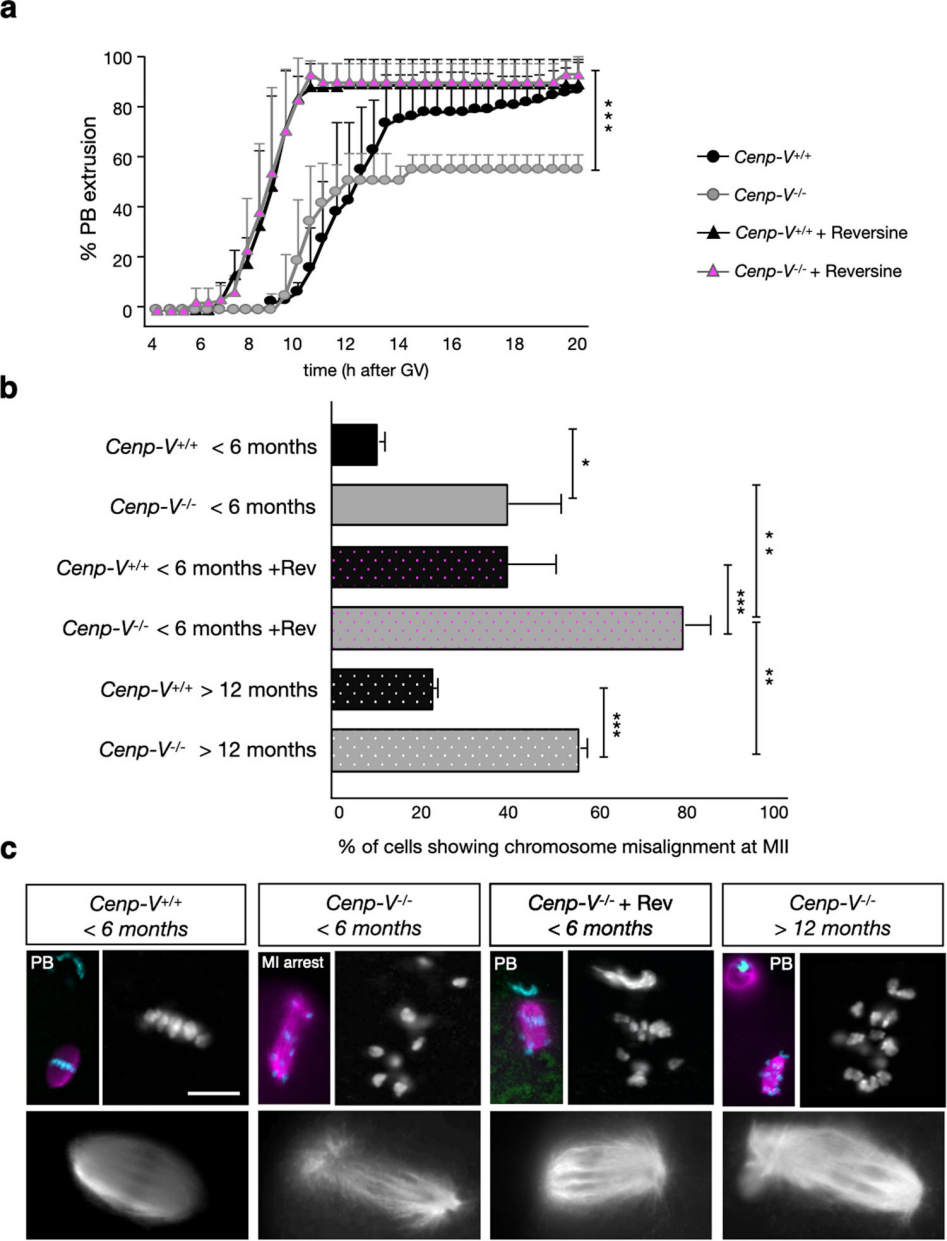
Inhibition of MPS I or ageing allow Cenp-V−/− oocytes to proceed to anaphase I. **a** Cumulative percentage of first polar body extrusion (PBE) over time in *Cenp-V*^*+/+*^ *and Cenp-V*^*−/−*^ oocytes treated with reversine. Note that the addition of reversine restores PBE to control values. The addition of reversine accelerates and increases PBE in both *Cenp-V*^*+/+*^ *and Cenp-V*^*−/−*^ oocytes. **b** Percentage of cells that harbour misaligned chromosomes at MII in young oocytes treated with reversine and in old cells. Reversine increases the misalignment also in control cells compared to non-treated cells. 4a: statistical differences refer to the average of all data points. Statistic: (**a**,**b**) data were expressed as mean ± SD and statistical differences were tested by a two way Anova test (***p < 0.001, **p < 0.01, *p < 0.05). The exact *p* values are available in the source data file. Statistical differences refer to the average of all data points. *Cenp-V*^*+/+*^ *and Cenp-V*^*−/−*^ oocytes treated with reversine show not a significant difference. (**a**) *n* = 112 oocytes from 3 independent experiments and (**b**) *n* = 10 cells per condition from 4 independent experiments. **c** Example of *Cenp-V*^*+/+*^ *and Cenp-V*^*−/−*^ oocytes treated with reversine in young females and one example of *Cenp-V*^*−/−*^ oocyte >12 months for comparison. Note that the absence of CENP-V in young females arrests half of the cells in MI and only after the addition of reversine all oocytes proceed to anaphase and extrude the PB. Microtubules are stained by anti-ß-tubulin (magenta), centromeres are stained with anti ACA (green) and DNA is stained by Hoechst 33258 (cyan). Enlarged images in grey shown the single channel of the chromosomes and spindle. Scale bar = 10 µm.

The reduction in PBE in the absence of CENP-V correlates with chromosome misalignment, which is the likely cause of oocyte arrest at MI. Interestingly, we observed a decrease in PBE only in oocytes from young females, but not in oocytes from females 12 months or older. We speculated that the SAC increasingly fails to prevent anaphase in ageing oocytes carrying chromosome and/or spindle aberrations. An age-dependent decline in SAC stringency has been previously suggested^2,5,29,30^. We hence tested if the SAC is responsible for the arrest of young *Cenp-V*^*−/−*^ oocytes before PBE. Thus, we inhibited the SAC kinase Mps1 by adding reversine to the culture medium of *Cenp-V*^*−/−*^ and *Cenp-V*^*+/+*^ oocytes at the GV stage^31^ (Fig. 4). PBE in reversine-treated control oocytes was accelerated and PBE in *Cenp-V*^*−/−*^ cells was fully restored to control values. This suggests that the SAC arrests *Cenp-V*^*−/−*^ oocytes of young females at metaphase I. Analysis of chromosome mis-alignment in reversine-treated oocytes from young adults revealed a significant increase. This indicates that upon SAC inhibition many more oocytes carrying misaligned chromosomes proceed to PBE and MII (Fig. 4b). Notably, unlike young *Cenp-V*^*−/−*^ oocytes, aged *Cenp-V*^*−/−*^ oocytes proceeded to PBE like controls (Fig. 2g). In the aged *Cenp-V*^*−/−*^ oocytes a strongly increased frequency of cells carrying misaligned chromosomes was observed (Fig. 4b). The absence of MI arrest in aged oocytes despite this presence of mis-aligned chromosomes confirms that the SAC is not as active anymore in oocytes from old females. Thus, CENP-V deficiency reveals age-dependent weakening of the SAC in oocytes.

### CENP-V maintains spindle integrity in vivo and binds, diffuses along, and bundles microtubules in vitro

It has been suggested that the absence of CENP-V disturbs microtubule dynamics^21^. A failure in microtubule dynamics could lead to problems in chromosome capture and alignment. Thus, we asked whether the lack of CENP-V affects oocyte spindle integrity. *Cenp-V*^*−/−*^ oocytes were fixed and stained for microtubules at different time points; at 7 h after GV to study the recently formed MI spindle and between 10-16 h to study the late MI spindle after arrest and after the spindle has reached the cortex. At late metaphase I, between 10-16 h of culture, 46% of *Cenp-V*^*−/−*^ oocytes but virtually no control oocytes showed aberrant spindles suggesting that the lack of CENP-V indeed disrupts spindle integrity (examples are shown in Fig. 4c and Sup. Figure 5a). In those cells that still show a defined spindle shape, the spindle pole width was measured. We observed that lack of CENP-V leads to loss of the canonical barrel-shaped spindles as the poles are less defined, diffusely widened, in *Cenp-V*^*−/−*^ oocytes (Sup. Figure 5b).

**Fig. 5:**
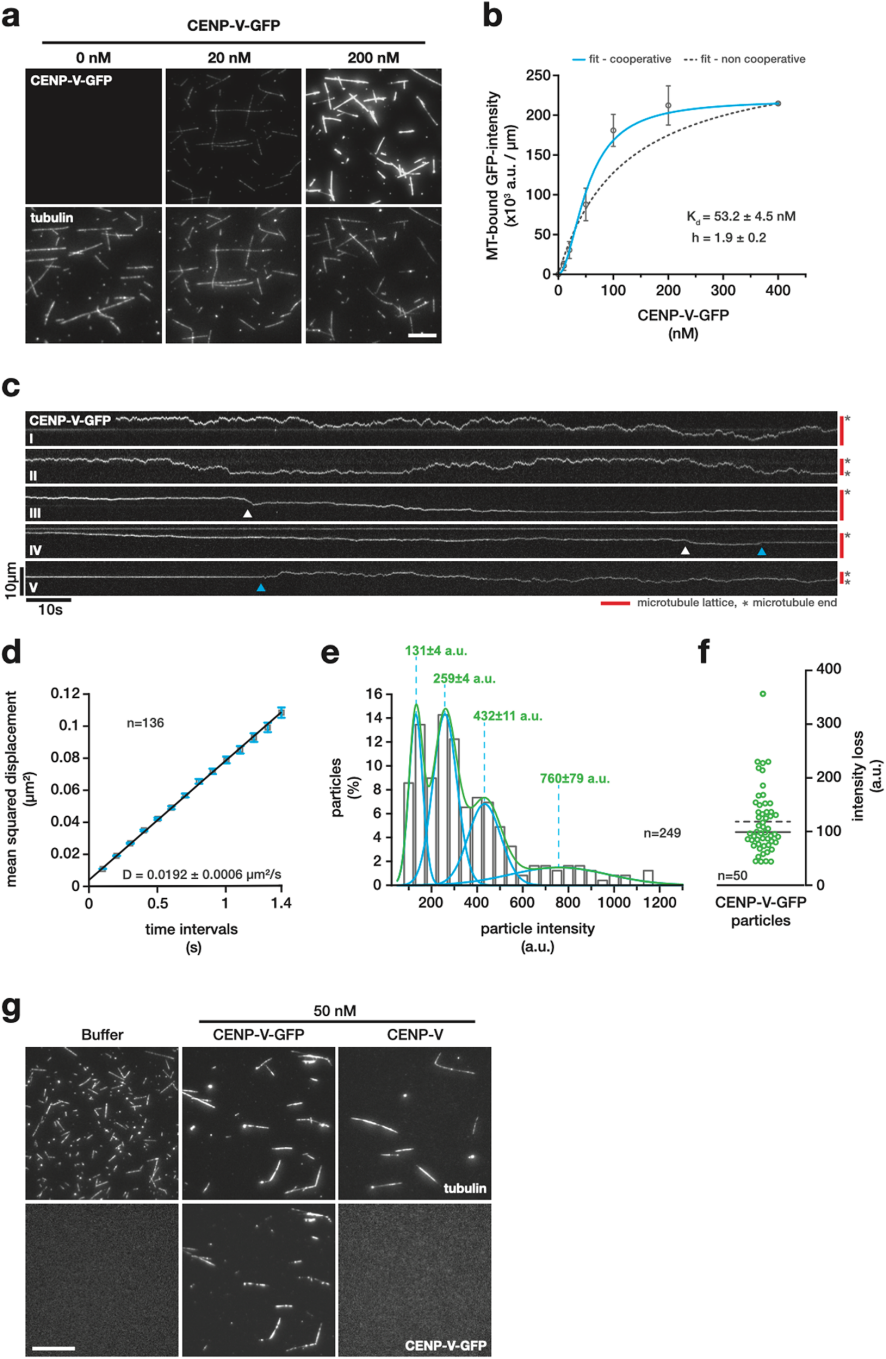
Multimers of CENP-V bind with high affinity to, diffuse along and bundle microtubules. **a** Binding of recombinant CENP-V-eGFP (*top panel row*) to taxol-stabilised ATTO 647N-labelled microtubules (*bottom panel row*) at the indicated CENP-V concentrations. Please refer to Sup. Figure 7A for pictures of the full concentration range and supplementary. Mov. S5. *Bar* equals 10 µm. **b** Saturation binding curve of CENP-V-eGFP showing the concentration-dependent fluorescence intensity of microtubule-associated CENP-V-eGFP in arbitrary units (arb.units) per micrometer. The dissociation constant (K_d_) and the Hill-coefficient (h) are given. *Error bars* indicate the standard error of the mean of three independent experiments. *Blue line* is a Hill-, *dashed grey line* a Michaelis-Menten-model fit. (**c**) Representative kymographs (*I-V*) showing the behaviour of 1 nM CENP-V-eGFP on the lattice of taxol-stabilised ATTO 647N-labelled microtubules in BRB80 at 25 °C. *Blue arrowheads* indicate transitions between static and motile periods. *White arrowheads* indicate fast shift events during slow diffusion episodes. *Scheme to the right* indicates the extend of the respective microtubule lattice (*red*) and the microtubule ends (*asterix*). (**d**) Mean square displacement (MSD) analysis of motile CENP-V-eGFP particles. Data is fitted with a linear regression (*black line*); *blue error bars* are the standard error of the mean. (**e**) Histogram showing the initial fluorescence intensity of motile CENP-V-eGFP particles – i.e., the mean intensity during the first (in average75) movie frames. *Blue lines* indicate Gaussian fits to the multiple peaks of the histogram adding up to the cumulative Gaussian fit shown as a *green line*. *Dashed lines* indicate the local maxima of the respective fits. The respective peak intensity values in arbitrary units (a.u.) ± standard error of the mean are given above. (**f**) Plot showing the intensity loss during single bleaching events (*n* = 50) of motile CENP-V-eGFP particles (*n* = 25). *Solid* and *dashed lines* indicate median and mean (98.9 a.u. and 118.3 a.u. ± 8.2 s.e.m. respectively). (**g**) Surface immobilised axol-stabilised ATTO 647N-labelled microtubules after pre-incubation with either buffer, 50 nM CENP-V or CENP-V-eGFP (*top panel row*). The corresponding eGFP-channels are shown in the *bottom panel row*. Scale bar = 20 µm. Representative images of three independent experiments are shown.

As previous work suggests that CENP-V can bind microtubules on its own^22^, we hypothesised that CENP-V-microtubule interactions might directly stabilise the acentrosomal meiotic spindle. To test this hypothesis, we expressed recombinant His_6_-CENP-V-eGFP in insect cells and purified the fusion protein by immobilised metal affinity and size exclusion chromatography (Supp. Figure 6). Biophysical properties of CENP-V were studied in vitro. We first quantified the microtubule-binding capacity of CENP-V-eGFP, by titrating increasing amounts of protein onto surface-bound, taxol-stabilised ATTO 647N-labelled microtubules in 80 mM PIPES, pH 6.8, 1 mM MgCl_2_, 1 mM EGTA (BRB80) and visualising the interaction by total internal reflection fluorescence (TIRF) microscopy (Fig. 5a, Sup. Figure 7A, B and Sup. Mov. S5). The binding of CENP-V-eGFP to the microtubule lattice was quantified by calculating the average GFP-intensity per micrometer for at least 100 microtubules per condition for three independent technical replicates. The resulting binding saturation curve was best fitted with a cooperative binding (Hill) model, suggesting that binding of CENP-V to the microtubule lattice is cooperative (Fig. 5b). Binding of CENP-V-eGFP occurred with a K_D_ of 53.2 ± 4.5 nM Fig. 5b), while single microtubule-associated particles were already evident at 1 nM (Fig. 5a–c).

To characterise the lattice behaviour of single CENP-V-eGFP particles, we next recorded three-minute time-lapse movies at 100 milliseconds time resolution, visualising the movement of 1 nM CENP-V-eGFP in BRB80 and at 25 °C on surface-bound, taxol-stabilised ATTO 647N-labelled microtubules by TIRF microscopy. In general, CENP-V-eGFP particles stayed attached to the microtubule lattice, with only a few associations or dissociation events to be observed within up to fifteen minutes (Fig. 5c, supplementary Mov. S5, supplementary Fig. 8A, B). Lattice-bound CENP-V particles can be forced to dissociate from the microtubule by an excess of free tubulin - but not by BSA - indicating that the CENP-V-tubulin interaction is specific (Sup. Fig. 8C). Particles showed complex motility on the microtubule lattice that can be described either by i) continuous, rather “fast” diffusion (Fig. 5c I, II), ii) very slow diffusion (Fig. 5c III, IV) with interdispersed short but fast diffusing episodes (Fig. 5c, *white arrowheads*) or iii) a mixture thereof, where CENP-V particles switch between modes. Furthermore, motile particles eventually became stationary for extended periods on the microtubule lattice (Fig. 5c IV, *blue arrowhead* to end of kymograph, switch motile to stationary and Fig. 5c V, beginning of kymograph to *blue arrowhead*, switch stationary to motile) or remained associated with the microtubule end (Sup Fig. 8A). At microtubule intersection, CENP-V-eGFP particles occasionally switched microtubule tracks and explored both microtubules (Sup Fig. 8D). A mean squared displacement analysis including all CENP-V-eGFP particles that showed some episodes of movement (*excluding* stationary episodes at microtubule ends or microtubule intersections) during the duration of the time-lapse movie, produced a rather low apparent diffusion coefficient of *D* = 0.0192 ± 0.0006 µm s^−1^ (*n* = 136) (Fig. 5d). As expected from the complex CENP-V motility on the microtubule lattice (Fig. 5C), however, the diffusion coefficient of single CENP-V-eGFP particles are distributed over at least one order of magnitude with maximum values of *D* = 0.15 µm s^−1^ (supplementary Fig. 8E).

Interestingly, motile CENP-V-eGFP particles also showed a rather broad intensity distribution with four local maxima at 131 ± 4, 259 ± 4, 432 ± 11 and 760 ± 79 arbitrary units (a.u., *n* = 249) (Fig. 5e). A comparison with the average intensity loss of 118 ± 8 a.u. (*n* = 50 bleaching steps from 28 particles) per GFP during bleaching events of these particles suggests that - in line with the observed cooperative binding behaviour - microtubule bound CENP-V can form multimers on the microtubule lattice (Fig. 5f, supplementary Fig. 9). Hence, we hypothesised that the broad distribution of diffusion coefficients might be a direct cause of the inhomogeneous particle size distribution. In this case, we would expect larger multimeric particles to interact stronger with the microtubule lattice causing slower diffusion rates compared to smaller particles. Subsampling of the data by grouping the particles in 125 arbitrary unit bins, however, showed that the distribution of diffusion coefficients does not change significantly with increasing particle size (Supplementary Fig. 8F). This suggests that the broad distribution of diffusion coefficients observed for single CENP-V particles is caused by peptide properties other than multimerisation.

The cooperative binding behaviour of CENP-V and its capacity to multimerise (on the microtubule lattice) is reminiscent of other microtubule-associated proteins like PRC1/Ase1 that structurally reinforce the mitotic spindle by bundling interpolar microtubules in antiparallel overlaps of the spindle midzone^32–36^. We hence asked, whether also CENP-V has the capacity to drive microtubule bundle formation. To test this, we incubated taxol-stabilized ATTO 647N-labelled microtubules with 50 nM CENP-V-eGFP or non-GFP-tagged CENP-V for 15 m at room temperature, immobilized them on the surface of glass flow-cambers, and flushed out non-bound CENP-V prior to imaging. Indeed, both 50 nM CENP-V and CENP-V-eGFP induced the formation of large microtubule bundles that were absent from the buffer-only control (Fig. 5g).

In summary, we conclude that CENP-V is a *bona fidae* microtubule-associated protein that binds cooperatively to the microtubule. It remains attached to the microtubule for a prolonged period exploring it by diffusion and it forms multimers that are able to cross-link and bundle microtubules. It therefore shows all characteristics of a structural MAP like PRC1/Ase1. This could explain the loss of spindle integrity we observed in CENP-V deficient oocytes in vivo. Thus we measured microtubule bundling in the meiotic spindle (Sup. Fig. 10). To remove unstable microtubules, MI ocytes were cold-treated before fixation. Microtubules were visualised by anti-Tubulin-GFP antibody. The spindle was divided in five equal regions and the thickness of the most and least prominent microtubule in each region was measured and defined as a maximum and minimum level of bundling^37^. We observed a reduction in both, maximal and minimal microtubule thickness, i.e. bundling, in *Cenp-V*^*−/−*^ oocytes (Sup. Fig. 10a). The maximum level of bundling in *Cenp-V*^*−/−*^ oocyte spindles does not even reach the minimum grade of bundling in controls. The overall level of spindle fluorescence was also reduced in *Cenp-V*^*−/−*^ cells compared to the control oocytes (Sup. Fig. 10b). The biochemical and oocyte data are consistent with impaired bundling causing the loss of spindle integrity. Next, we asked whether CENP-V’s function extends to microtubule-chromosome interactions.

### CENP-V is required for proper chromosome-microtubule attachment in metaphase I

**Fig. 6:**
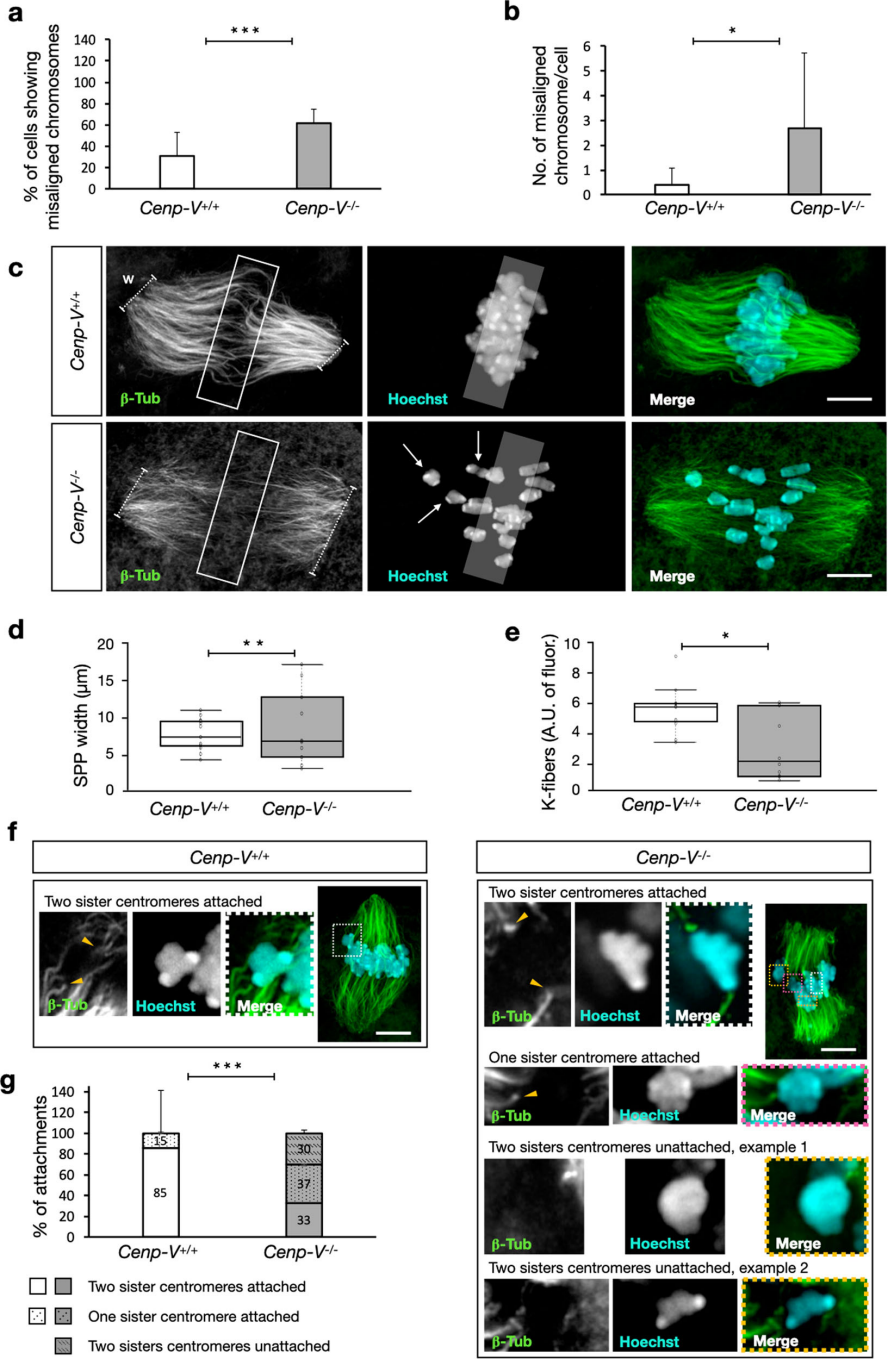
Metaphase I spindle and chromosome-microtubule attachment in fixed oocytes after 10 h culture and cold treatment. **a** Percentage of cells showing chromosome misalignment. Chromosome misalignment is defined when the chromosome is entirely outside the region of the equatorial plate (transparent area in c) (**b**) Average number of chromosome misalignments per oocyte. (**c**) Example of *Cenp-V*^*+/+*^ and *Cenp-V*^*−/−*^ oocytes. Microtubules (left) and DNA (center) are stained by anti ß-tubulin and Hoechst 33258 respectively. See also supplementary Movies S6 and S7. Scale bar = 10 µm. (**d**) Spindle pole width (SPP width; w, dashed line in c) in *Cenp-V*^*++*^ and *Cenp-V*^*−/−*^ oocytes. (**e**) K-fiber quantification expressed as arbitrary units (A.U.) of fluorescence within the boxed region shown in **c**. (**f**) Confocal planes of chromosome-microtubule attachments in *Cenp-V*^*+/+*^ and *Cenp-V*^*−/−*^ oocytes. A maximum projection of the whole spindle is shown for each genotype. A proper attachment to two sister-centromeres is indicated by a white dashed square in *Cenp-V*^*+/+*^. For *Cenp-V*^*−/−*^ oocytes different types of attachments are shown: two sister centromere attachment (white); attachment to one sister centromere (pink); and unattached sister centromeres (yellow). The tip of the K-fiber attached to the centromere is indicated by a yellow arrowhead. Centromeres are identified as the strongest region stained by Hoechst 33258. Please see also supplementary Movies S8 and S9. Scale bar = 10 µm. (**g**) Quantification of chromosome-microtubule attachment. Statistic: data were expressed as mean ± SD and statistical differences were tested by a paired two tailed *T*- test (***p < 0.001, **p < 0.01, *p < 0.05). The exact *p* values are available in the source data file. Statistical differences refer to the average of all data points. (**d**, **e**) Data are represented as box plots where the middle line is the median, the lower and upper boxes correspond to the first and third quartiles and outlying points are plotted beyond the end of the whiskers. (**a**, **b**) *n* = 30 cells; (**d**) *n* = 21 cells, (**e**) *n* = 24 cells and (**g**) *n* = 113 attachments. All data come from 3 independent experiments.

It was suggested that the SAC control may be less stringent in oocytes than in somatic cells, not being able to recognize a single unattached kinetochore^5^. It is uncertain whether the presence of one or two mis-aligned chromosomes would be sufficient to alone trigger the SAC. Failure to properly attach kinetochores to the spindle may add to the signals triggering the SAC. Therefore, we decided to assess chromosome-microtubule attachment in fixed cells by super-resolution microscopy to determine the number of unattached chromosomes in young and old oocytes at metaphase I and II. Before fixation, oocytes were incubated in a cold buffer to preserve stable, kinetochore-attached microtubules (K-fibres), while the less stable, non-attached microtubules are lost. This treatment allows us to visualise specifically those K-fibres that are well attached to the chromosomes^38,39^. Because live imaging experiments (Figs. 2, 3) showed that in control cells at 10 h after GV almost all chromosomes are well aligned at the metaphase plate and the spindle is at the cortex but still ahead of anaphase and PBE, the attachments of homologous chromosomes were analysed in fixed oocytes after 8–10 h of maturation (Fig. 6). The levels of chromosome mis-alignment were similar to those in live cell experiments (Fig. 6a, compare to Fig. 3b), supporting our previous results and validating the time point of cell fixation. Again, a significantly higher number of *Cenp-V*^*−/−*^ cells showed chromosome mis-alignment. Each oocyte was imaged as a z-stack with a spatial resolution of 145 nm allowing us to distinguish between single homologous pairs. At this high resolution we counted almost 3 mis-aligned chromosomes per *Cenp-V*^*−/−*^ oocyte compared to less than 1 in controls (Fig. 6b and supplementary Mov. S6 and supplementary Mov. S7).

In oocytes the absence of centrosomes is functionally replaced by self-organizing microtubule organizing centres (MTOCs) that form de novo from a cytoplasmic microtubule network leading to the formation of the spindle and its polarity^40,41^. Since we observed CENP-V colocalizing with MTOCs and with the spindle (Fig. 1 and Supplementary Fig. 2), β-TUBULIN staining was used to measure the spindle pole width (SPP width) as an indicator of spindle pole integrity (Fig. 6c, d). It is important to consider that in this experiment all oocytes are used for quantification although at 7-10 h of culture it is not possible to determine which oocytes will later extrude the PB and which will not. Further, at this time point one cannot predict which spindles become compromised. Therefore, although all values are represented in the box plot, only those values of *Cenp-V*^*−/−*^ bigger than the median value in *Cenp-V*^*+/+*^ are considered for statistic differences. We observed that *Cenp-V*^*−/−*^ spindles show increased variability in SPP width and often increased SPP width, i.e. there is a lack of spindle pole integrity in *Cenp-V*^*−/−*^ spindles. To discard the possibility that the observed phenotype was due to an unspecific effect of the cold treatment, the SPP width was also measured in non-cold treated cells yielding the same results (Sup. Fig. 5b). A central region at the equatorial plane was defined to quantify the K-fibers as arbitrary units of fluorescence of the tubulin channel within that region (Fig. 6c). Cold-treatment removed the instable non-K-fibers. *Cenp-V*^*−/−*^ spindles showed a dramatic reduction in K-fibres: the control spindles showed 3 -fold more K-fibers than the *Cenp-V*^*−/−*^ spindles (Fig. 6e).

Lastly we analysed the mode of microtubule attachment to the homologous chromosomes. Three different types of attachments were identified: (i) two sister centromeres attached by microtubules from different poles, i.e. the correct attachment to properly segregate the homologous chromosomes in anaphase I; (ii) one sister centromere attached by microtubules from one pole; and (iii) both sisters centromeres unattached. Indeed, we found entire homolog pairs unattached (selected examples are shown in Fig. 6f, Supplementary Mov S8, and Supplementary Mov. S9). About 30% of the homologous chromosomes in *Cenp-V*^*−/−*^ oocytes were unattached, while there were no unattached chromosomes in control cells. Of the remaining 70% of chromosomes, almost half showed a wrong attachment in absence of CENP-V with only one sister centromere attached. In control cells, only 15% wrong attachments were observed in total (Fig. 6g). We tested the possibility that wrong or entirely missing attachments are a consequence of a perturbed centromere structure, as described for HeLa cells where CENP-V was reduced^20^. Therefore, sister centromere distances were measured in *Cenp-V*^*−/−*^ and *Cenp-V*^*+/+*^ metaphase I chromosome spreads from non cold-treated, unperturbed cells, and no differences in centromere distance were found between both genetic backgrounds (Sup. Fig. 11). This indicates that sister centromere cohesion is not affected, consistent with our earlier results of lack of single unpaired chromatids in metaphase II.

Taken together these experiments show that CENP-V plays an important role in kinetochore-microtubule attachments and is required for proper metaphase I chromosome alignment and spindle formation.

### CENP-V is required for correct chromosome-microtubule attachment during metaphase II

**Fig. 7:**
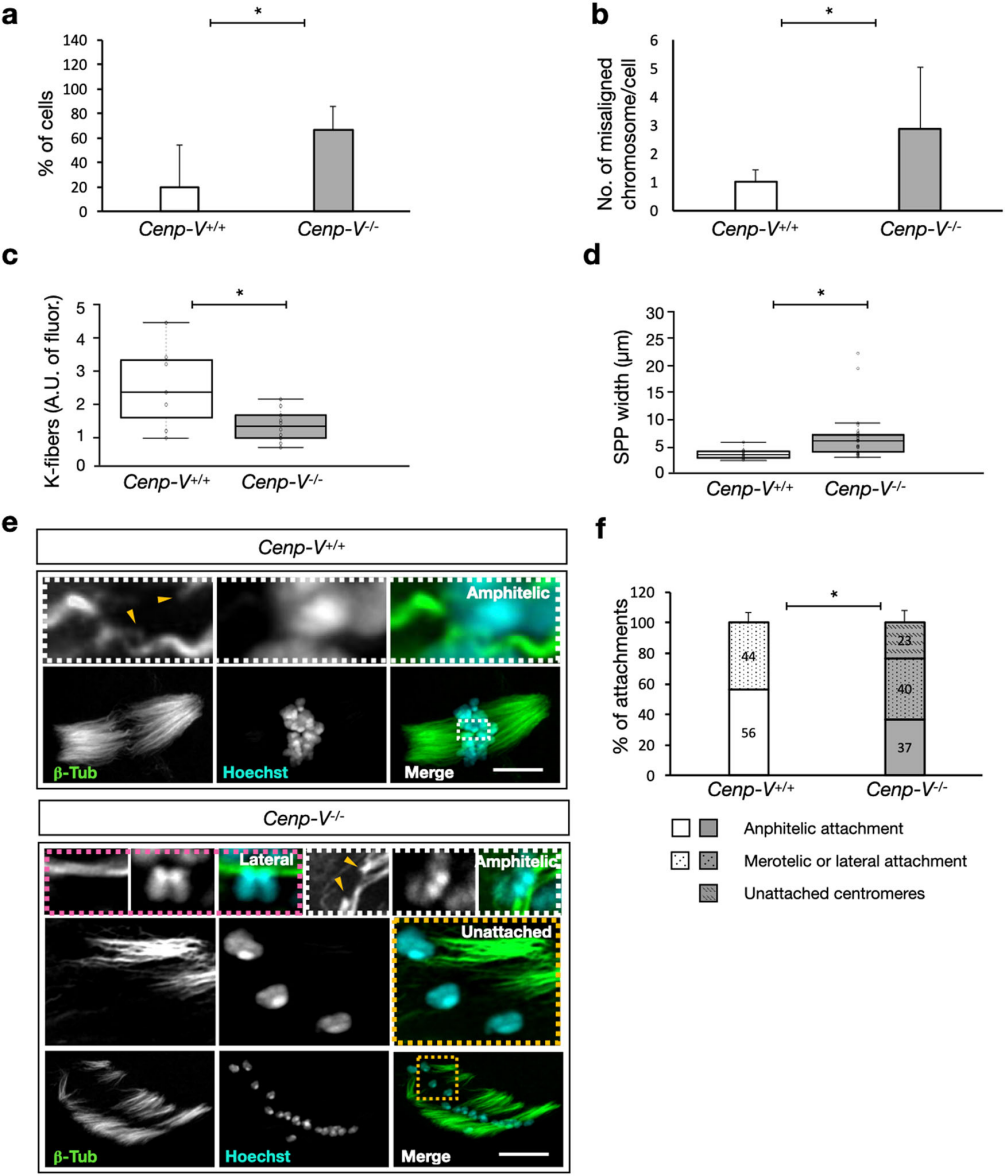
Metaphase II spindle and chromosome-microtubule attachments in oocytes fixed 5-7 h after polar body extrusion and cold treatment. (**a**) Percentage of cells showing chromosome misalignment. (**b**) Average number of chromosome misalignments per cell. (**c**) K-fiber quantification expressed as arbitrary units (A.U) of fluorescence (**d**) Spindle pole width (SPP width, w) quantification. (**e**) Example of chromosome-mt attachments in *Cenp-V*^*+/+*^ and *Cenp-V*^*−/−*^ oocytes. A maximum projection of the whole spindle is shown for each genotype. Single planes of z-stacks are shown for every attachment. One example of a proper amphitelic attachment to the centromeres is shown for *Cenp-V*^*+/+*^ *and Cenp-V*^*−/−*^ oocytes, indicated by a white dashed square. A merothelic or lateral attachment and three examples of unattached sister centromeres are shown for *Cenp-V*^*−/−*^ oocytes, pink and yellow dashed square respectively. Microtubules are stained by anti ß-tubulin and DNA stained by Hoechst 33258. The tip of the K-fiber attached to the sister centromere is indicated by a yellow arrow head. Centromeres are identified as the strongest region stained by Hoechst 33258. Images were taken on an Airyscan confocal microscope, the z plane distance was 145 nm. See also supplementary Movies S10 and S11. Scale bar = 10 µm (**f**) Chromosome-microtubule attachment quantification. Statistic: data were expressed as mean ± SD and statistical differences were tested by a paired two tailed *T*- test (***p < 0.001, **p < 0.01, *p < 0.05). The exact p values are available in the source data file. Statistical differences refer to the average of all data points. (**c**,**d**) Data are represented as box plots where the middle line is the median, the lower and upper boxes correspond to the first and third quartiles and outlying points are plotted beyond the end of the whiskers. (**a**, **b**, **c**, **d**) *n* = 54 cells and (**f**) *n* = 79 attachments. All data come from 3 independent experiments.

Since erroneous MT-chromosome attachments in metaphase II (MII) could lead to aneuploidy as well, we decided to take advantage of the fact that old *Cenp-V*^*−/−*^ oocytes progress to metaphase II. We performed cold treatment to visualize the attachments at MII in aged *Cenp-V*^*−/−*^ oocytes (>12 months). To be sure the cells have reached the MII arrest, we fixed the cells 5 h after PB extrusion as published^39^, and performed quantifications as for MI (Fig. 7).

Like in MI, in MII we found a strong difference in chromosome alignment at the equatorial plate in *Cenp-V*^*−/−*^ oocytes: proper alignment in only 33% of the cells compared to 80% in control oocytes; Fig. 7a). The number of mis-aligned sister chromatids per cell was also higher in *Cenp-V*^*−/−*^ oocytes with more than 3 sister chromatids per cell, in some cells up to 5 mis-aligned sister chromatids were found (Fig. 7b). Following the same criteria as in the previous experiment (Fig. 6c) the K-fibers and the polarity of the spindle were quantified. In the absence of CENP-V there is a significant app. 2-fold reduction of K-fibers and a moderate loss of spindle polarity (Fig. 7c, d). As described in^39^ we found a high number of merotelic or lateral attachments in old control and *Cenp-V*^*−/−*^ oocytes. However, we did not find any pair of sister chromatids unattached in controls compared to 23% of unattached sister chromatid pairs in *Cenp-V*^*−/−*^ oocytes (Fig. 7e, f, Supplementary Mov. S10 and Supplementary Mov. S11). This is consistent with data shown in Fig. 2 on strongly increased aneuploidy in *Cenp-V*^*−/−*^ oocytes.

Thus, as in MI, CENP-V contributes importantly to MII spindle integrity, chromosome attachment, and chromosome alignment.

## Discussion

Here we report that the poorly described protein CENP-V crucially contributes to mammalian meiosis I and II. In meiotic prophase I CENP-V accumulates at and near the centromeres. In oocytes, which feature a unique mode of spindle assembly through acentrosomal MTOCs, CENP-V localises to these MTOCs and gradually associates with the spindle when the oocytes mature, i.e. progress through meiosis I up to metaphase II. This spatial distribution is in line with data presented in the three papers describing CENP-V in somatic cells where it localises to the kinetochores, the mitotic spindle midzone, or other microtubule structures in the leading edge of migrating cells or primary cilia^20–22^. The appearance of CENP-V starting in zygotene suggests that CENP-V, which is typically expressed in somatic cells and thus in premeiotic cells, vanishes at the entry into meiosis and is only later expressed again. The reason for this disappearance is unknown, but it indicates the rapid turnover of the protein.

Independently of oocyte age, the absence of CENP-V in oocytes results in aberrant spindle formation and in a significantly, up to a five-fold increased level of chromosome misalignment with associated subfertility and aneuploidy. Several of the results such as meiotic resumption data were obtained not only from the constitutive CENP-V deficiency model but also from three germ cell-specific Cre-driven conditional CENP-V deficiencies demonstrating that the phenotypes observed are germ cell-intrinsic and not indirectly caused by deficiencies of surrounding somatic cells. CENP-V deficient spermatocytes however, did not display an obvious phenotype. As oocytes and spermatocytes assemble their spindle by different mechanisms, we hence propose that CENP-V is dispensable for the conventional centrosome-based mechanism used in spermatocytes. In contrast to spermatocytes oocytes perform asymmetric cell division leading to the exclusion of the polar body. This process involves elaborate movement of the spindle to the cell cortex. In this context, CENP-V could render the spindle more resilient to mechanical stress during its relocation.

The resumption of oocyte meiosis and thus GVBD does not require CENP-V. This suggests that the preceding steps of early oogenesis do not depend on CENP-V. This hypothesis is supported by our observation of identical phenotypes in all CENP-V deficiency models, regardless of whether constitutive or induced in early prophase or shortly after birth, i.e. in dictyate arrested oocytes. To trigger the phenotypes described in this communication it is sufficient to induce CENP-V deficiency in these arrested oocytes and thus shortly before resumption of meiosis. For successful PBE, the chromosomes need to be all properly aligned on the metaphase plate, the spindle must be formed, including kinetochore fibers that must attach at the kinetochores. These processes are impaired in absence of CENP-V.

Spindle microtubules can be divided into three major classes: kinetochore microtubules, which form parallel k-fibers ending at the kinetochore; interpolar microtubules, which extend from the opposite poles of the spindle and form antiparallel overlaps in the spindle midzone as well as astral microtubules, which extend from the poles towards the cell cortex. Recent work in human somatic cells^42^ has shown a close relationship between interpolar and kinetochore microtubules, where interpolar bundles are attached laterally to k-fibers almost all along their length, acting as a bridge between k-fibers of sister kinetochores. Thus, the spindle is made of modules consisting of a pair of sister kinetochore fibers and a bundle of interpolar microtubules that connects them. These interpolar bundles, termed bridging fibers, balance the forces acting at kinetochores and support the shape of the spindle during metaphase^43,44^. In absence of CENP-V, the acentrosomal spindle poles are not focused properly and k-fibers become irregular and unstable shortly before PBE. We hence hypothesize that loss of CENP-V weakens the force-balancing capacities of the kinetochore- and bridging-fibre-system thereby reducing the force resilience of the meiotic spindle. Weakened structurally, the spindle might disintegrate during its migration to the cortex leading to non-attached and misaligned chromosomes.

In line with this, our biophysical characterisation identifies CENP-V as a genuine microtubule associated protein that is functional reminiscent of structural MAP prototypes like PRC1/Ase1. Like PRC1/Ase1, CENP-V shows cooperative binding to the microtubule in the moderate to low nano molar range (K_D_ = 53 ± 5 nM vs. 5.5 nM for Ase1^36^ and 600 ± 300 nM for PRC1 MTBD^35^, it diffuses rather slowly on the microtubule lattice (D = 0.0192 ± 0.0006 µm^2^/s vs. 0.055 ± 0.005/0.085 ± 0.007 µm^2^/s for Ase1^33,36^ and shows a high microtubule residency time in the minute range (vs. approximately 10 s for Ase1 and PRC1). CENP-V has, like Ase1/PRC1, the ability to dimerise or multimerise, which is likely to be crucial to its co-operative binding behaviour and to its microtubule cross-linking activity, since CENP-V only harbours one microtubule domain, the zinc-finger domain at amino acids 162–227^21^. Due to its capacity to efficiently bundle microtubules and due to its functional similarity to PRC1/Ase1, we propose that the primary role of CENP-V in the meiotic spindle is that of a microtubule bundler, which helps to cross-link and focus microtubules at the acentrosomal spindle poles. Additionally, CENP-V might increase the mechanical resilience of the spindle by cross-linking and stabilising k-fibres and their bridging bundles, thereby preventing spindle breakage during cortex translocation. Based on its biophysical properties it is likely that CENP-V also contributes by other means, like the modulation of microtubule dynamics, to meiotic spindle stability, which warrants further mechanistic studies on this protein.

CENP-V deficient oocytes of young adults are strongly impaired in PBE, i.e. PBE is delayed and happens in only app. half of the oocytes. Administering a SAC inhibitor allowed *Cenp-V*^*−/−*^ oocytes to perform PBE like the control indicating that the SAC is activated and responsible for the PBE failure. Surprisingly, oocytes from aged mice performed PBE like the controls despite the continuous presence of mis-aligned chromosomes and aberrant spindles, suggesting that the SAC is not as active in these aged oocytes. Thus, CENP-V reveals age-dependent weakening of the SAC. Our findings therefore support the suggestion of SAC weakening as another important contributor to age-dependent oocyte failure, i.e. chromosome mis-segregation and thus aneuploidy^2,13,29^. This process comes in addition to well-established mechanisms, particularly loss of sister chromatid cohesion. We did not observe single, unpaired sister chromatids in absence of CENP-V. Thus, centromeric sister cohesion itself is not lost in this mutant, rather proper segregation of pairs of sister chromatids is failing. When age-dependent cohesion loss happens its consequences are likely exacerbated by weakening of the SAC since a fully functional SAC would prevent oocytes that suffer cohesion loss from proceeding. Therefore, we suggest a cumulative effect of cohesion loss and SAC weakening as important drivers of age-dependent aneuploidy. This model does not exclude other mechanisms such as telomere damage-induced chromosome fusions that should also trigger the SAC, likely to fail in old oocytes.

The absence of CENP-V causes a failure to properly capture and align chromosomes and thus has a very significant biological consequence. Ageing CENP-V deficient oocytes, despite carrying abnormal numbers of chromosomes, underwent meiosis I up to metaphase II as the SAC failed. This contributes to either subfertility or to aneuploid embryos, some of which may survive, or both. Assuming that these basic mechanisms and processes are conserved between mouse and man, this is of obvious concern for human reproductive health.

## Methods

### Isolation, culture and live imaging of mouse oocytes

All mice were bred and maintained under a 12 h light/12 h dark cycle at 22 ^o^C (± 2 ^o^C) and 55 % humidity (± 5 %) in the animal facility of the medical faculty, Technische Universität Dresden, according to approved institutional guidelines. All animal experiments were performed according to the relevant ethical requirements and were approved by the state animal welfare office (Dr. B. Langen; license numbers TVA03/2016; TVV51/2016; TVA07/2021).

In our study, we used 6–12 weeks old C57BL/6 females except for ageing studies in which 12–14 months old mice were used. Ovaries were isolated in M2 medium (Sigma) supplemented with 250 μM milrinone (Sigma) and punctured with a 27 gauge needle to release the oocytes. GV stage oocytes were collected with mouth pipetting and maintained at prophase arrest in M2 with milrinone under paraffin oil (Sigma) at 37 °C. For analysis of GVBD and polar body extrusion oocytes were released into M2 medium and imaged every 30 min for up to 20 h on an inverted Nikon TE2000E microscope with a Plan APO 20 ×/0.75 NA objective, a CoolSNAP HQ camera (Photometrics), standard filter sets, and with an environmental chamber to maintain 37 °C. Z-series optical stacks of 80 µm were recorded with slices taken every 7 μm. All images were acquired using the Nikon NIS-Elements ND2 software and analyzed with imageJ. Where indicated, reversine was added at 6 h after GVBD at a final concentration of 500 nM. Because it is soluble in oil, reversine was also added to the oil covering the media drops.

### Generation of *Cenp-V* knockout mouse strain

To generate a germ cell specific CENP-V deficiency, we have obtained three clones of ES cells in which Exon 3 of the gene is flanked by loxP sites to be recombined by Cre recombinase. The clones (*Cenpv*^*tm1a(EUCOMM)Hmgu*^) were received from EUCOMM, the relevant strain information can be found here: https://www.mousephenotype.org/data/genes/MGI:1920389#order. An insertion cassette had to be deleted first by using FLP recombinase, which recombines the FRT sites. We have obtained highly (90 %) chimeric mice and have bred them with C57BL/6 mice. Germline transmission was obtained and Cenp-V^fl/+^ mice were bred to the Flpo transgenic strain expressing FLP recombinase. Cenp-V^fl/+neo-^ progeny was obtained and bread with germ cell specific Cre-expressing mouse strains. Excision of the floxed exon was confirmed by DNA sequencing. We used the following strains, all of which have been shown to efficiently delete target genes in germ cells(1) Vasa-Cre, which expresses Cre in late spermatogonia in males and at entry into meiosis in females;(2) GDF9-Cre, which expresses Cre in females at day 2 after birth, and(3) SPO11-Cre, where Cre is expressed in male and female early prophase.

### Oocyte chromosome spreads

Oocytes chromosome spreads were prepared according to an adapted method from Hodges and Hunt, 2002. The zona pellucida of oocytes arrested at metaphases I was removed using acid treatment, Tyrode’s solution, (T1788; Sigma), and oocytes were transferred onto one well of a 10-well glass slide into 15 μl of fixation solution (1% PFA, 0.1% Triton X-100, 3 mM DTT; pH 9.2). After drying overnight at room temperature, slides were washed 3 × 5 min in PBS and stained immediately.

### Immunofluorescence and image acquisition

For whole-mount immunofluorescence staining oocytes were cultured in M2 plus or minus 2.5 μM milrinone and collected at the relevant time points. The zona pellucida was removed using tyrode’s solution. After a 30 min recovery period, the oocytes were fixed in 2% formaldehyde and 0.1% Triton X-100 in M2 media for 10 min. Cells were washed once in blocking buffer (3-5% bovine serum albumin (BSA) and 0.1% Triton X-100 in phosphate-buffered saline (PBS)), before incubation in blocking buffer for 30 min at room temperature. The cells were transferred into the primary antibody solution (3% BSA and 0.1% Triton X-100) and incubated at 4 °C overnight. Primary antibodies used were as follows: rabbit anti-CENP-V (HPA042616; Sigma) at 1:100, rabbit anti-CENP-V (homemade) at 1:250, rat anti-α-tubulin (MCA78G; Bio-Rad) at 1:5000, mouse anti-SYCP3 (clone 60C10) a gift from Dr. C. Heyting at 1:500, mouse anti-γ-tubulin (ab11316; Abcam) at 1:5000, human anti-ACA (ANI-15-235-000; Biozol) at 1:50, goat anti-CENP-A (sc-11277; Santa Cruz) at 1:100. Secondary antibodies were used as follow: anti-rabbit Alexa Fluor 488 (A27034; Invitrogen) at 1:500, anti-mouse Alexa Fluor 647 (715-605-151; Jackson Immuno Research) at 1:500, anti-mouse Alexa Fluor 568 (715-605-151; Invitrogen) at 1:500, anti-human Alexa Fluor 568 (A-21090; Invitrogen) at 1:500, anti-sheep Alexa Fluor 568 (A-21099; Invitrogen) at 1:500, anti-goat Alexa Fluor 488 (ab150129; abcam). The cells were washed at RT in 0.1% Tween 20 in PBS 3 × 5 min and mounted in Vectashield (H-1000; Vector Laboratories) plus 1 μg/ml DAPI (4′,6-diamidino-2-phenylindole) for imaging. Fixed oocytes were imaged with an inverted Zeiss Axio Imager Z2 microscope using a 63 x/ 1.4 NA oil objective. Brightness and contrast of the images were modified for presentations.

### In vitro transcription, microinjection and oocyte live imaging

*β-tubulin-Gfp*, histone *H2b-Rfp*, and *Cenp-V-Gfp* mRNAs were synthesized with the T3 or T7 mMessage mMachine Kit (Ambion) according to the manufacturer’s instructions and purified using RNAeasy columns (Qiagen). The plasmids pRN3–GFP–β-tubulin and pRN3-histone H2B-RFP were a gift from K. Wassmann (CNRS, Paris, France; Touati *et al*., 2012). Mouse *Cenp-V-Gfp* was cloned into pOCC13 (Max Planck institute CBG, Dresden) plasmid and mRNA was transcribed and purified as above.

Oocytes arrested in prophase were injected using a FemtoJet microinjector (Eppendorf) with constant flow settings. To allow expression of fusion proteins and oocyte recovery, oocytes were incubated for 2 hr in M2 media supplemented with milrinone. Where tubulin was recorded 100 nM SiR-tubulin final concentration was added in the M2.

After release into M2 medium, oocytes were imaged with Nikon TE2000E microscope with a Plan APO 20 ×/0.75 NA objective. Airyscan images were taken using the airyscan module on a laser scanning microscope (LSM880, Zeiss) with a plan APO 63 ×/ 1.4 NA oil objective.

To define the localization of CENP-V respect to the spindle we applied Pearson’s colocalization coefficient (r) for the spindle pole and for the spindle center and measured the colocalization between the -CENP-V-GFP signal and SiR Tubulin along the z-stacks using FIJI software. This value represents the percentage of CENP-V that colocalizes with SiR Tubulin. Perfect colocalization is considered when this value is close or equal to 1 and no colocalization when is close or equal to 0.

### Expression and purification of recombinant CENP-V

CENP-V was fused to an N-terminal His-Tag and/or monomeric GFP, expressed from a baculovirus vector in SF9 insect cells (Expression Systems, cat# 94-001 F, https://expressionsystems.com/product/insect-cells/). Three days after infection, cells were harvested and lysed in buffer A (20 mM Hepes, 1 M NaCl, 20 mM imidazole, 1 mM DTT, pH 7.5) supplemented with EDTA-free Protease Inhibitor Cocktail (Roche) using an Emulsiflex C5 (Avestin). The lysate was cleared by centrifugation (18.000 g, JA-25.50 rotor (Beckman Coulter), 60 min, 4 °C). Soluble material was applied to a HisTrap 5 ml column (GE Healthcare) and washed with 10 column volumes (CV) buffer A, 5 CV ATP-buffer (5 mM ATP in 2xPBS 10 mM MgCl_2_) and with buffer A containing 40 mM imidazole. Finally, the protein was eluted by applying the elution buffer (20 mM Hepes, 1 M NaCl, 300 mM imidazole, 1 mM DTT, pH 7.5). The eluted fraction was concentrated by centrifugation using an Amicon concentrator (10 kDa cutoff), and subjected to size exclusion chromatography using a Superdex-200 16/60 column (GE Healthcare) equilibrated with 20 mM Hepes, 300 mM NaCl, pH 7.0 at room temperature, using an Akta Pure chromatography system (GE Healthcare). Pooled samples were concentrated by centrifugation in Amicon tubes and snap frozen in liquid nitrogen.

### TIRF microscopy

Total internal reflection fluorescence (TIRF) microscopy experiments in this study were performed on an Axio Observer Z1 microscope (Carl Zeiss Microscopy GmbH, Jena, Germany), equipped with 488 nm/532 nm / 642 nm laser lines (Omicron-Laserage, Rodgau-Dudenhofen, Germany) and an iXon X3 EMCCD (Andor Technology, Belfast, UK). Image data were acquired using an alpha Plan-Apochromat 63x/1.46 Oil objective (Zeiss) with a 1.6-fold auxiliary magnification - achieving a final pixel size of 159 ± 3 nm. For temperature control, the objective was equipped with a water-supplied heating-ring connected to a thermostatic circulator (F-25-MC, Julabo, Seelbach, Germany).

### Microtubule binding assays

For the microtubule-binding assays, taxol-stabilised Atto647N-labelled microtubules were grown at 37 °C for one hour in 80 mM PIPES, pH 6.8, 1 mM MgCl_2_, 1 mM EGTA, pH 6.8 (BRB80) supplemented with 4 mM MgCl_2_, 1 mM GTP and 4.8% (v/v) DMSO. Microtubules were sedimented at room temperature for 30 m at 17.000 g and resuspended in warm BRB80 supplemented with 10 µM taxol (Sigma). Anti-β tubulin antibodies (mouse, SAP.4G5, Sigma) at 15 µg/ml were allowed for 10 m to bind to the silylated glass surface of a flow cell. The surface then was blocked for one hour with 1% Pluronic F-127 (Sigma). Surplus Pluronic F-127 was washed out using BRB80 and Atto647N-labelled microtubules were allowed to bind to the anti-β tubulin antibodies until the desired density was reached. CENP-V-eGFP was diluted in assay buffer (BRB80, 10 μM taxol, 50 μg⋅ml–1 casein, 10 mM DTT, 20 mM glucose, 0.2 mg⋅ml^−1^l–1 glucose oxidase (Serva Electrophoresis GmbH, Heidelberg, Germany), and 20 μg⋅ml–1 catalase (Sigma)) to reach the desired concentration. The flow channels were perfused with CENP-V-eGFP at the respective concentrations in assay buffer and sealed with (vaseline/lanoline/paraffine, 1:1:1 wt/wt/wt).

In the microtubule binding assay, CENP-V-eGFP was pre-diluted in 20 mM HEPES, pH 7.2, 300 mM NaCl followed by a final dilution step in assays buffer to reach the indicated concentrations. Prior to imaging, the sealed flow chambers were allowed to equilibrate for 15 m at 25 °C on the microscope. Then, snapshots of the GFP- (488 nm laser line at 5 mW laser power, 100 ms exposure) and the tubulin-tubulin (642 nm laser line at 5.6 mW laser power, 300 ms exposure) were taken at random places. To minimise experimental variations, a full set of concentrations was recorded on the same day using the same stock solutions in flow channels on the same glass coverslips. The integrated total intensity of microtubule-bound CENP-V-eGFP and the length of individual microtubules were measured in ImageJ. Microtubule sections with intersecting or overlapping microtubules were not considered for analysis. For each condition, the mean intensity per micrometer was calculated and corrected for the chamber-specific background mean values off the microtubules as well as for the microtubule-specific background in the 488 nm channel (empty buffer control). Binding curve data was fitted with a Michaelis-Menten model (y = B_max *_ x/(K_D_ + x)) or a Hill model (y = B_max *_ x^h^/(K_D_^h^ + x^h)^) using Origin 2019.

For the diffusion analyses of CENP-V-eGFP, the protein was diluted to 1 or 5 nM in assay buffer and flowed into the imaging chamber. Sealed chambers were allowed to equilibrate for 5 m on the microscope at 25 °C. Then a snapshot of the microtubule positions (642 nm laser line at 5.6 mW laser power, 300 ms exposure) was taken followed by either a 3 m (continuous stream at 100 ms exposure) or a 15 m (timelapse at one frame per second) movie of the GFP channel using the 488 nm laser line at 5 mW laser power. Tracking of single CENP-V-eGFP particles and calculation of the diffusion coefficient D were carried out by FIESTA v1.6^45^ using the following settings (alterations from standard settings only): threshold - relative intensity, height, 1; connecting - maximum velocity, 5000 nm⋅s–1; weights, 100% position; tracks - minimum length, 10 frames; maximum break, 10 frames; maximum angle, 180°. The resulting tracks were manually validated based on the CENP-V-eGFP signal in the corresponding kymographs. Kymographs were produced in ImageJ using the MultipleKymograph plugin (www.embl.de/eamnet/html/body_kymograph.html). The initial intensity of motile CENP-V-eGFP particles corresponds to the median particle intensity during the first approximately 75 frames of the movie as derived from a linescan along the particle trace in the corresponding kymograph using ImageJ. This approach was chosen to compensate for a minor frame to frame intensity fluctuations.

For the forced dissociation experiment, Atto647N-labelled taxol-stabilised microtubules were preloaded with 10 nM CENP-V-GFP in assay buffer and equilibrated for 3 m at 25 °C on the microscope. Then a snapshot of the microtubule position was taken followed by a 10-m time-lapse movie of the GFP-channel at 2 frames per second-time resolution using the laser setting described for the diffusion analyses above. Approximately four m into the movie, the 488 nm laserine was transiently switched off and the chamber was perfused with 10 nM CENP-V-GFP and 4 µM (unlabelled) free tubulin or bovine serum albumin (BSA) as crowding control.

For the bundling assay, taxol-stabilised microtubules were incubated with or without CENP-V(-eGFP) at the indicated concentration and incubated for 15 m at room temperature. The mix was perfused into a flow chamber and the microtubule (-bundles) were allowed to attach to the surface bound antibodies for 10 m. Unbound microtubules and CENP-V-eGFP were washed out with assay buffer, the chambers sealed and imaged as described for the microtubule-binding assay.

### Cold treatment and Airy scan imaging

Microtubules were stabilized by fixation in 1.9% formaldehyde in BRB80 buffer (80 mM PIPES, 1 mM MgCl_2_, 1 mM EGTA, pH 6.8) after 5 min cold treatment in 80 mM PIPES, 1 mM MgCl_2_, pH 7.4, as described in^46^. To obtain oocytes at metaphase II, cells were fixed after 6-8 h after the first polar body extrusion as^39^. Images with kinetochore–MT attachments were collected at the Zeiss 800 with an Airyscan module using the 63×/1.4 NA objective. The z plane distance was 145 nm. The spindle axis was placed through the centre, and the spindle equator plane was perpendicular to the spindle axis and crossed it at the centre. A central region in the equatorial plane with a thickness of 100 pixels was used to quantify the fluorescence of microtubule forming K-fibers. Any chromosome entirely outside the region of the equatorial plate was considered misaligned. DNA was stained by Hoechst 33258 and centromeres were considered the strongest stained region in the chromosome. Two parallel lines to the equatorial plane were drown into the poles to measure the spindle pole width. Images were processed by ZEN blue software with the Airyscan processing module and Fiji^47^.

### Statistics and reproducibility

The data processing, statistical analysis, and plotting was performed in Fiji^47^, MATLAB (Bitplane), Excel (^©^ 2015 Microsoft), and GraphPad Prism (GraphPad Software, Inc.). Error bars indicate means ± standard deviation (SD). Sample sizes and statistical tests are indicated in the figure’s legends. All experiments were performed a minimum of three times.

### Reporting summary

Further information on research design is available in the Nature Research Reporting Summary linked to this article.

## Supplementary information

Supplementary Information

Description of Additional Supplementary Files

Supp. Movie 1

Supp. Movie 2

Supp. Movie 3

Supp. Movie 4

Supp. Movie 5

Supp. Movie 6

Supp. Movie 7

Supp. Movie 8

Supp. Movie 9

Supp. Movie 10

Supp. Movie 11

Reporting Summary

## Data Availability

The data generated in this study has been deposited in the BioStudies database under accession code “S-BSST699”. A Source Data file is also provided. Accession to the nucleotide sequence of the floxed *Cenp-v* locus is available at https://www.eummcr.org/search?q=Cenpv&b=Go and http://www.informatics.jax.org/allele/MGI:4435304. All other relevant data supporting the key findings of this study are available within the article and its Supplementary Information files or from the corresponding author upon reasonable request. Source data are provided with this paper.

## Source data

Source Data
