# Supplementary material for: CENP-V is required for proper chromosome segregation through interaction with spindle microtubules in mouse oocytes
Source: Nat Commun. 2021 Nov 11;12:6547. doi: 10.1038/s41467-021-26826-3 (PMC8586017; doi:10.1038/s41467-021-26826-3)
Supplement: Supplementary file 1 — Supplementary Information [file 41467_2021_26826_MOESM1_ESM.pdf]

CENP-V is required for proper chromosome segregation through interaction with spindle microtubules in mouse oocytes

Supplemental Figure 1, Nabi et al

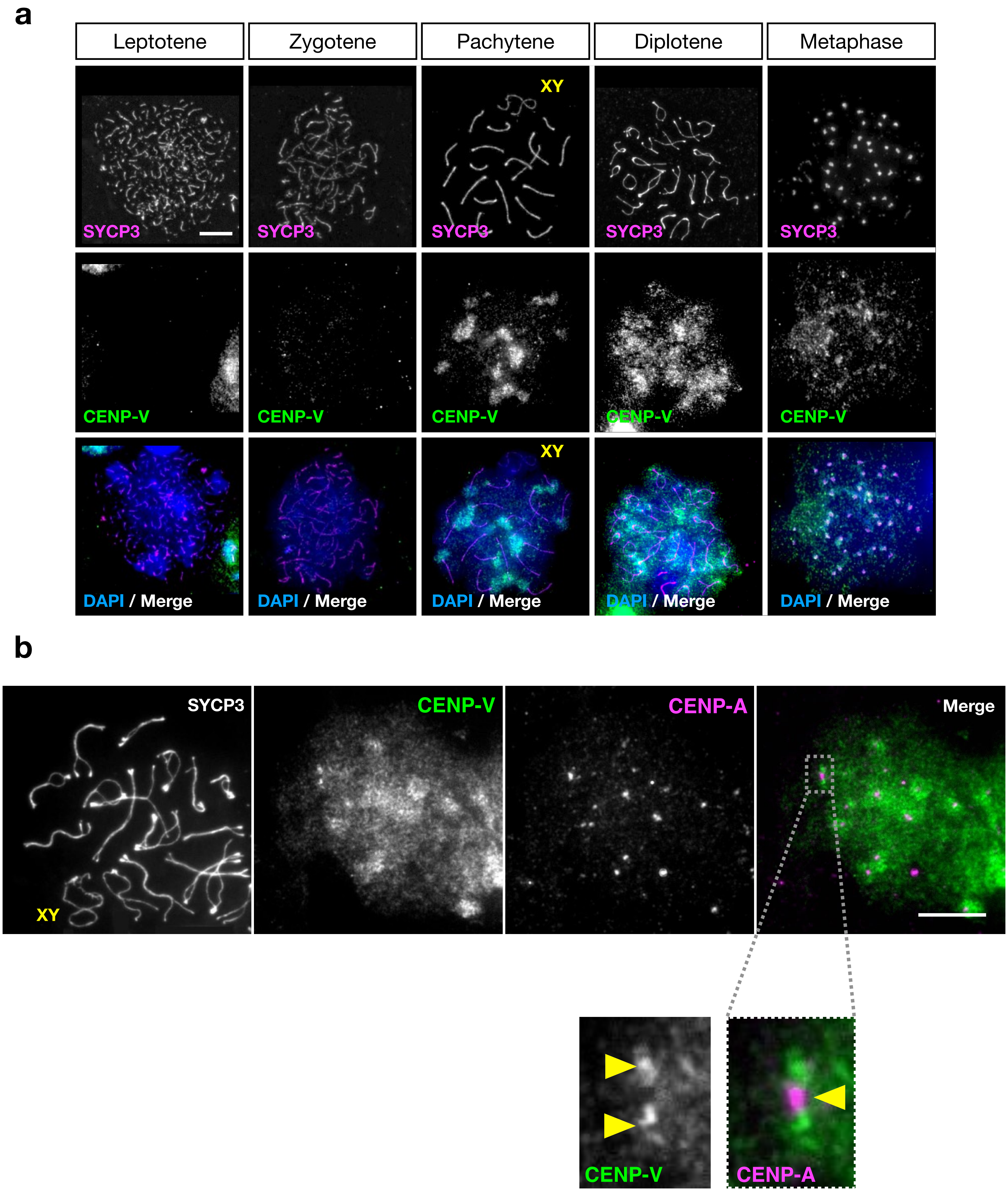

Supplemental figure 1: CENP-V localises to the centromere regions in male spermatocytes. (a) Spermatocyte chromosome spreads of *Cenp-V*$^{+/+}$ mice stained with anti-SYCP3 (magenta) for AEs/LEs and anti-CENP-V (green). CENP-V signal starts to appear as at zygotene and concentrates at the centromere regions from pachytene on. Note the lack of CENP-V in the sex body (XY); n=50 cells from 3 independent experiments. Scale bar = 5 µm. **(b)** Example of a diplotene cell from spermatocyte chromosome spreads of *Cenp-V*$^{+/+}$ mice stained with anti-SYCP3 (grey) for AEs/LEs, anti-CENP-V (green) and anti-CENP-A (magenta) for centromeres. Insets show higher magnification views of a centromere region. Note that CENP-V staining is close but external to CENP-A (yellow arrows). Scale bar = 5 µm.

Supplemental Figure 2, Nabi et al

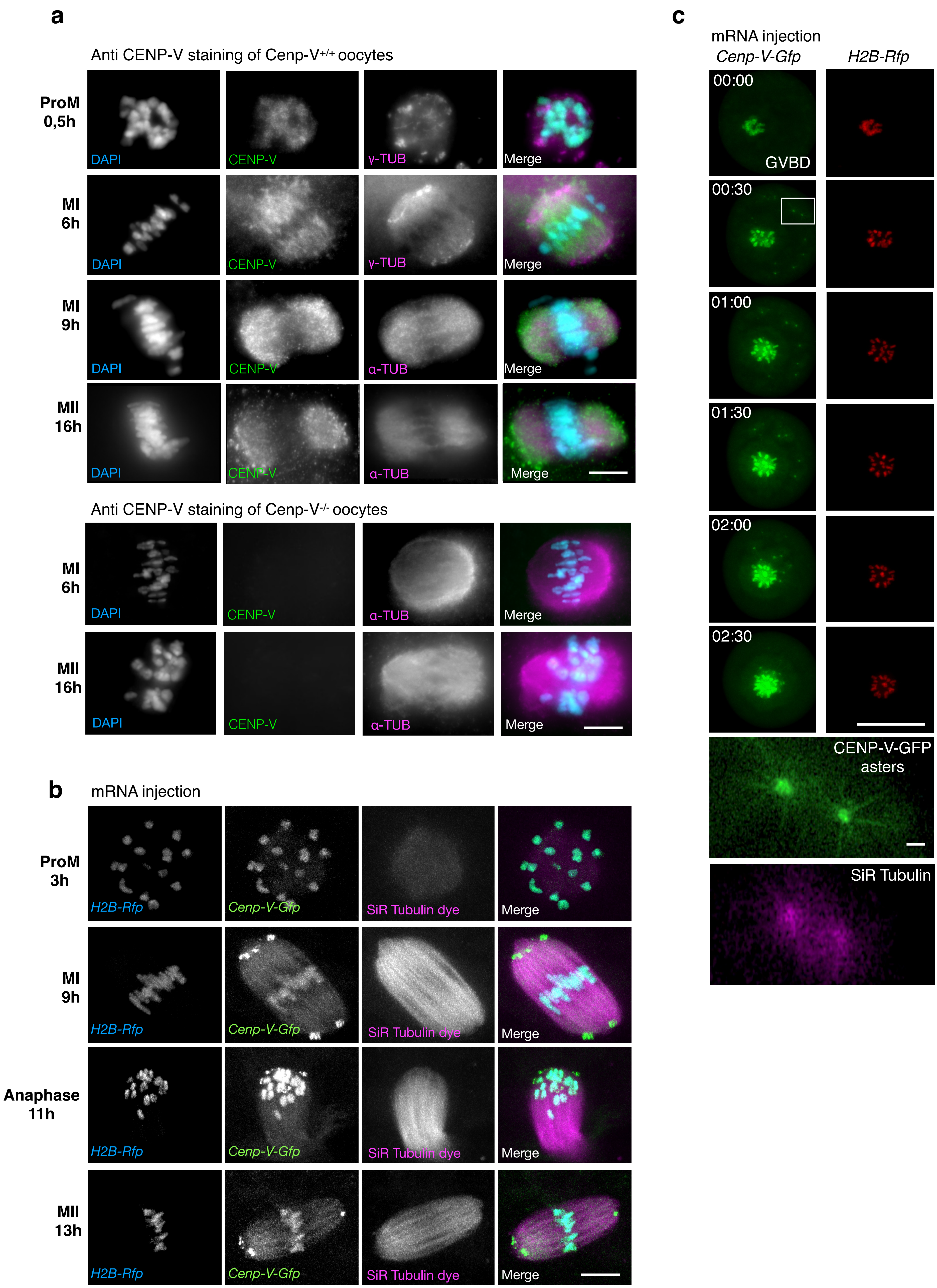

Supplemental Figure 2: CENP-V distribution during meiotic resumption. (a) CENP-V distribution during oocyte maturation in *Cenp-V*$^{+/+}$ (upper panel) and *Cenp-V*$^{-/-}$ (lower panel) whole oocytes by immunofluorescent staining using the indicated antibodies and DAPI. Note the absence of CENP-V signals in the *Cenp-V*$^{-/-}$ oocytes. Scale bar = 10 µm **(b)** CENP-V distribution during oocyte maturation in whole, fixed oocytes after injection of Cenp-V-Gfp mRNA. Note that the distribution of the CENP-V protein corresponds to that seen in a. **(c)** Time course of initial activation stages of oocytes injected with Cenp-V-Gfp mRNA. Scale bar = 20 µm. Note that at 1 to 2.5 hours after GVBD (enlarged images), CENP-V-GFP is seen in MTOC-like dots and also forms asters as seen in the bottom image, visualised by SiR Tubulin. Scale bar = 2,5 µm.

Supplemental Figure 3, Nabi et al

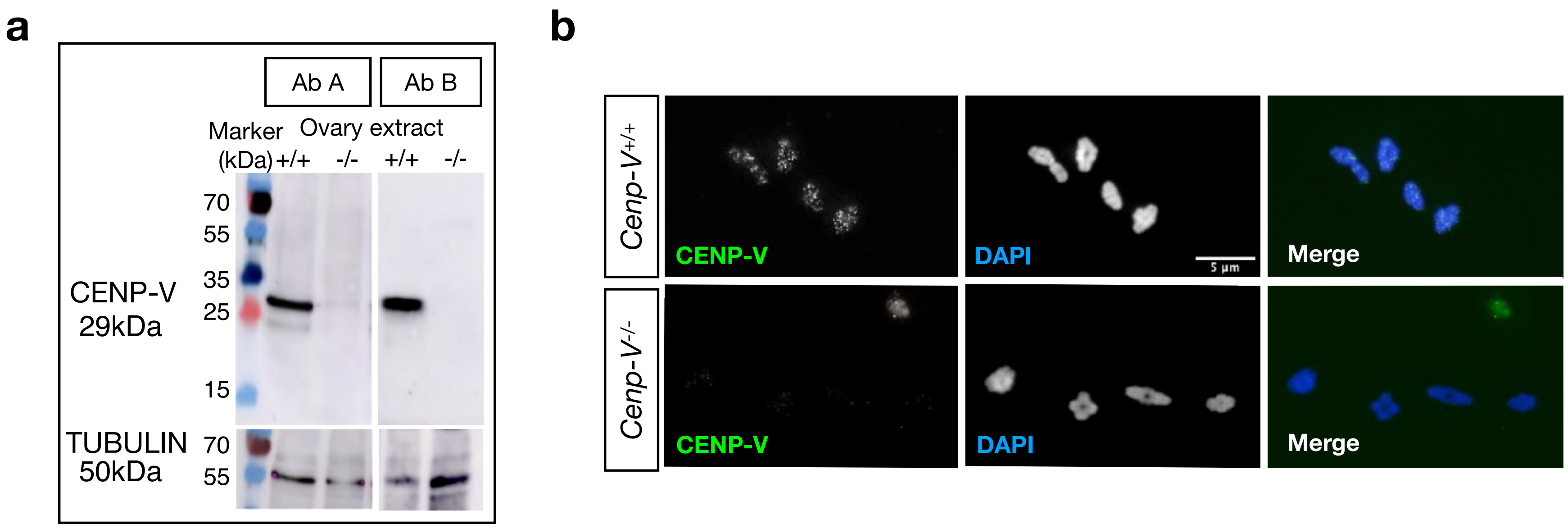

Supplemental figure 3: CENP-V deletion in *Cenp-V*$^{-/-}$ mice. (a) Protein levels of CENP-V and TUBULIN in *Cenp-V*$^{+/+}$ and *Cenp-V*$^{-/-}$ ovaries tested by Western blot (WB) with a comercial anti-CENP-V antibody from Sigma Inc. (A) and with an in-house produced anti-CENP-V antibody (B). Uncropped blots in Source Data. **(b)** Chromosome spreads at metaphase I of *Cenp-V*$^{+/+}$ and *Cenp-V*$^{-/-}$ oocytes stained with in-house produced anti-CENP-V (green) and DAPI (DNA). Scale bar = 5 µm. Commercial and in-house produced antibodies showed the same pattern in WB and chromosome spreads.

Supplemental Figure 4, Nabi et al

a

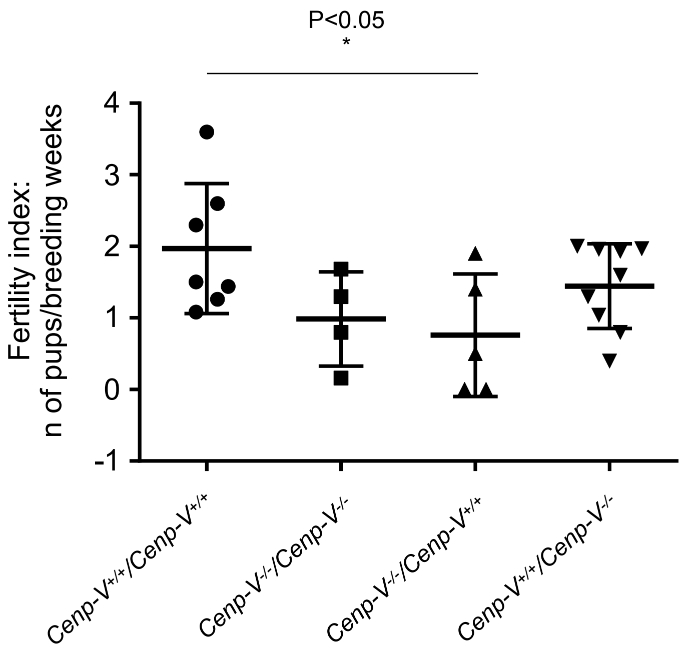

b

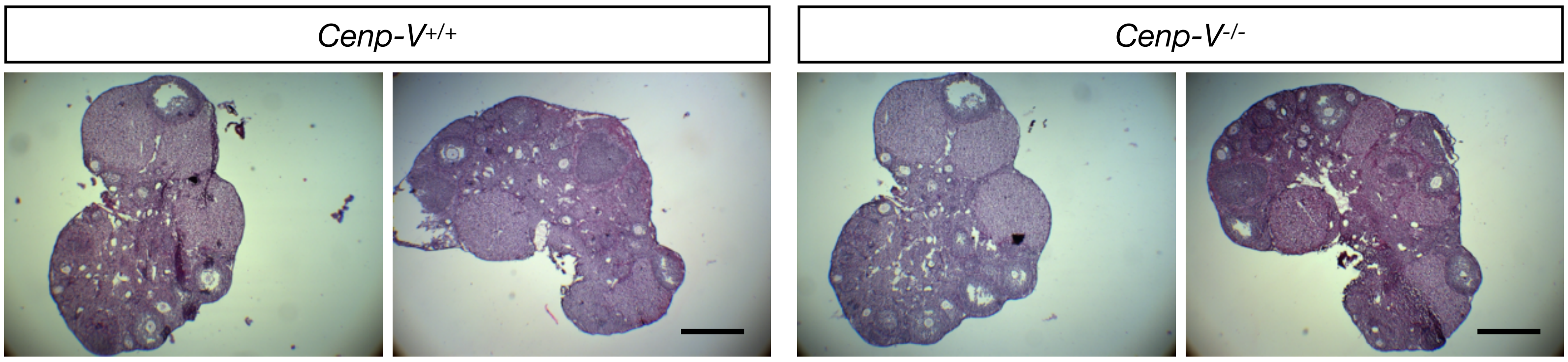

Supplemental figure 4: Subfertility of *Cenp-V*$^{-/-}$ mice and histology of their ovaries. (a) Dot plot showing the fertility index of the different breeding pairs. Data were expressed as mean ± SD. The first genotype is the female and the second is the male (female/male). Each dot represents a breeding pair. There is a significant difference between the fertility index of Ctrl/Ctrl and *Cenp-V*$^{-/-}$/Ctrl breadings. Statistical differences were tested by a paired two tailed T- test (****p* < 0.001, **p < 0.01 , *p < 0.05); the exact p values are available in the source data file. n = 25 breeding pairs **(b)** Histological sections of ovaries from *Cenp-V*$^{+/+}$ and *Cenp-V*$^{-/-}$ deficient mice. Entire ovaries were sectioned every 7 µm and stained with hematoxylin-eosin. *Cenp-V*$^{+/+}$ and *Cenp-V*$^{-/-}$ ovaries did not show any difference in the general architecture and follicular development. Scale bar = 0.5 mm.

Supplemental Figure 5, Nabi et al

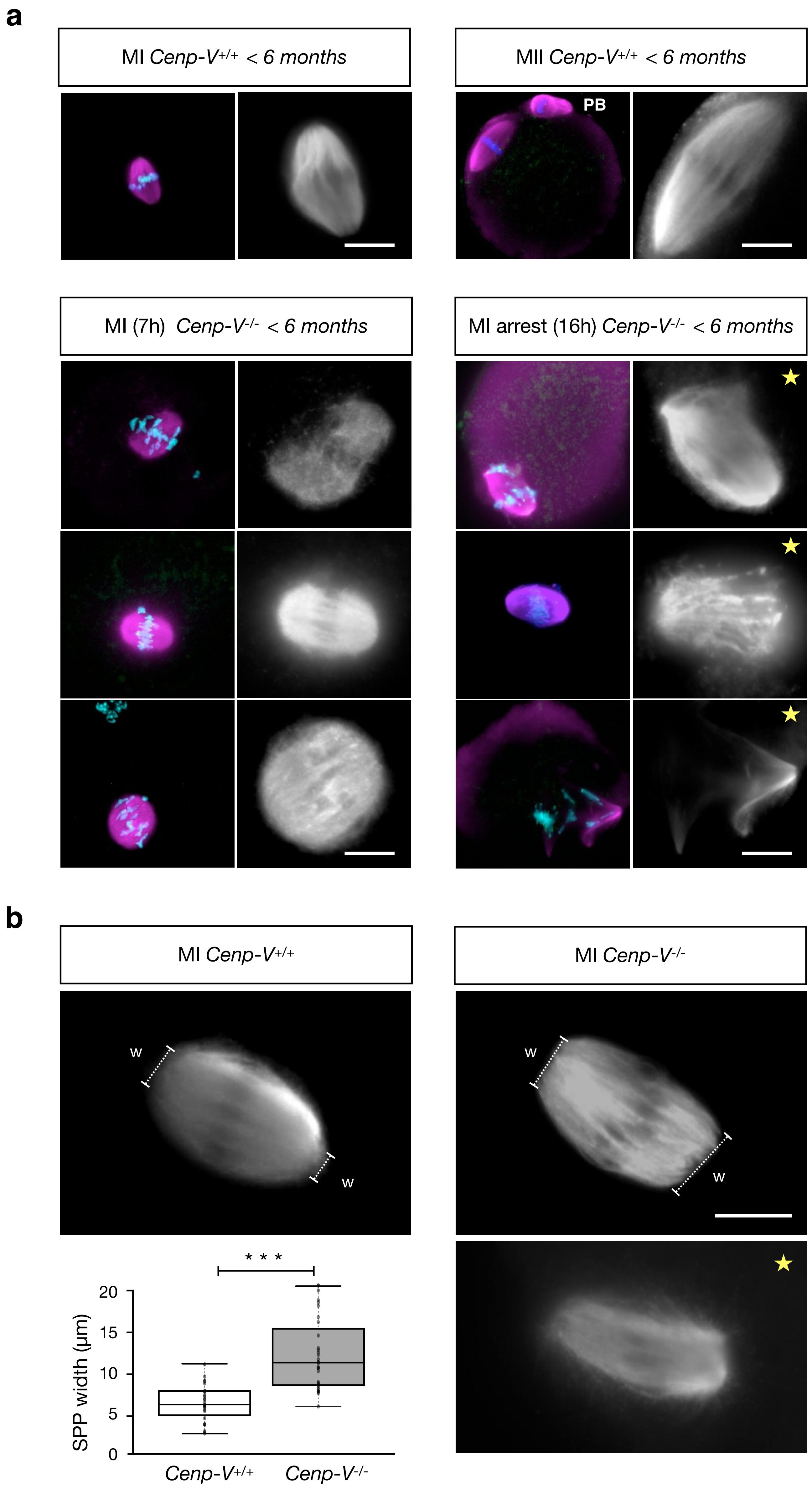

Supplemental figure 5. Aberrant spindle morphology in the absence of CENP-V. (a) Examples of *Cenp-V*$^{+/+}$ and *Cenp-V*$^{-/-}$ oocytes fixed after 7h or 16h culture. Microtubules are stained by anti **β**-tubulin, centromeres by anti ACA and the DNA is stained by Hoechst 33258. Note that as long as MI proceed the lack of CENP-V lead to the formation of aberrant spindles shapes (yellow stars) compared to the well defined bipolar spindle in control cells. We found 46 % aberrant spindles in the *Cenp-V*$^{-/-}$ oocytes arrested at MI compared to 2 % found in control. The spindle is considered aberrant when it shows more than two poles, extra microtubules reaching out of the poles and/or lack of bundles. We found aberrant spindles between 10-16 h of oocyte culture; scale bar= 10µm. n= 60 cells from 3 independent experiments. **(b)** Spindle pole width (SPP) analysis in non-cold treated cells. Two examples where the s**pindle pole width (w) is depicted by a dashed line are shown**. Scale bar= 10µm. Data are represented as box plots where the middle line is the median, the lower and upper boxes correspond to the first and third quartiles and outlying points are plotted beyond the end of the whiskers. n= 32 cells from 3 independent experiments. Statistical differences were tested by a paired two tailed T- test (****p* < 0.001, **p < 0.01 , *p < 0.05). The exact p values are available in the source data file. Outlier values were not included in the T-test. (a,b) Yellow stars indicate examples of aberrant spindles that are not include in the qualification of SPP width due to present unclear spindle poles and therefore considered aberrant spindles.

Supplemental Figure 6, Nabi et al

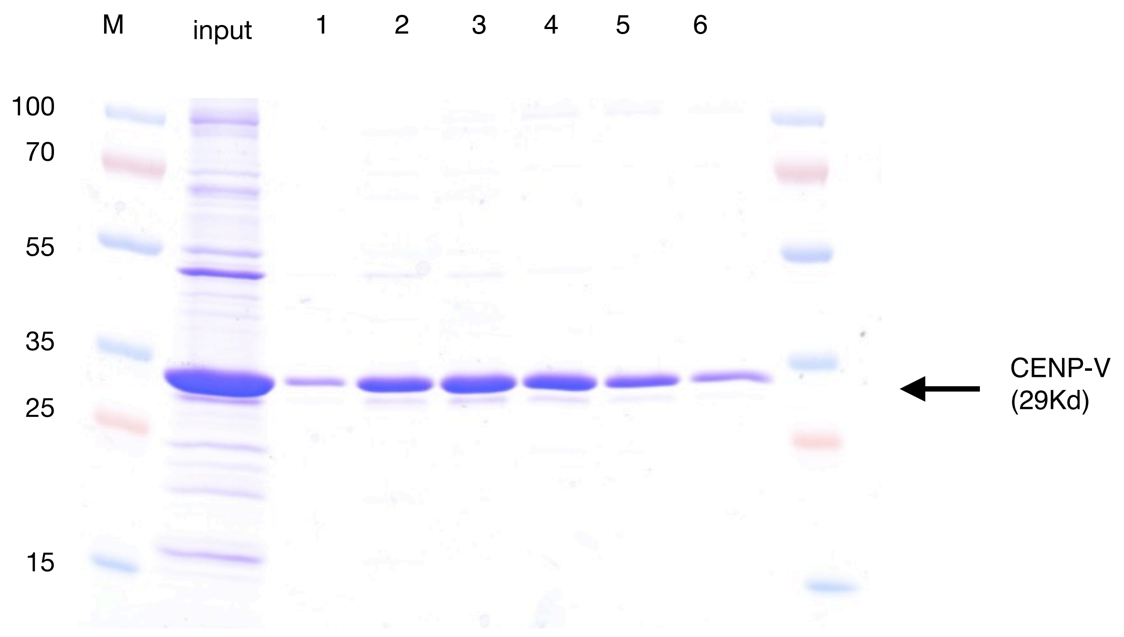

Supplemental Figure 6: CENP-V expressed by baculovirus in SF9 insect cells. SDS acrylamide gel showing consecutive fractions of the protein after size exclusion chromatography using a Superdex-200 16/60 column. Uncropped blots in Source Data. CENP-V appears as a single band of app. 29 kDa in SDS gel chromatography. The size exclusion fractions No. 1 to 6 correspond to molecular masses of about 220 to 50 kDa, with the majority of CENP-V eluting at about 180 kDa, indicative of a hexameric form. Lower mass forms such as those eluting at a dimer position were also observed.

Supplemental Figure 7, Nabi et al

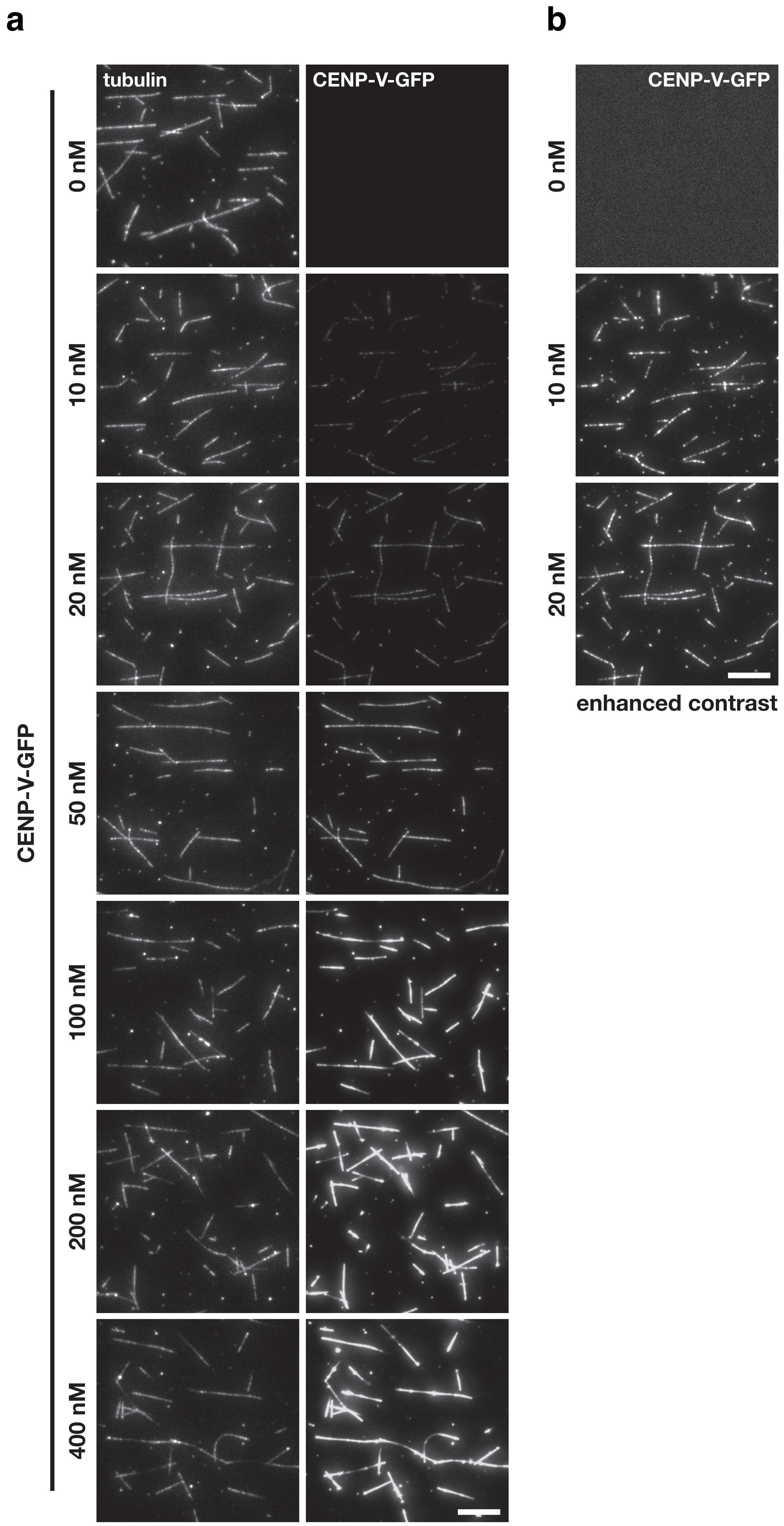

Supplemental Figure 7: Microtubule binding of CENP-V-eGFP. (**a**) Binding of recombinant CENP-V-eGFP (*right panel*) to taxol-stabilised ATTO 647N-labelled microtubules (*left panel*) at the indicated CENP-V concentrations. Pictures cover the full concentration range used to generate the saturation binding curve of CENP-V-eGFP shown in Fig. 5B. Please note that the tonal grey-scale range is constant across the concentration range. Enhanced contrast versions (i.e. pushed background) of the low concentration panels (0 - 20 nM CENP-V-eGFP) are shown in (**b**). Scale bar = 10 µm in (a) and (b).

Supplemental Figure 8, Nabi et al

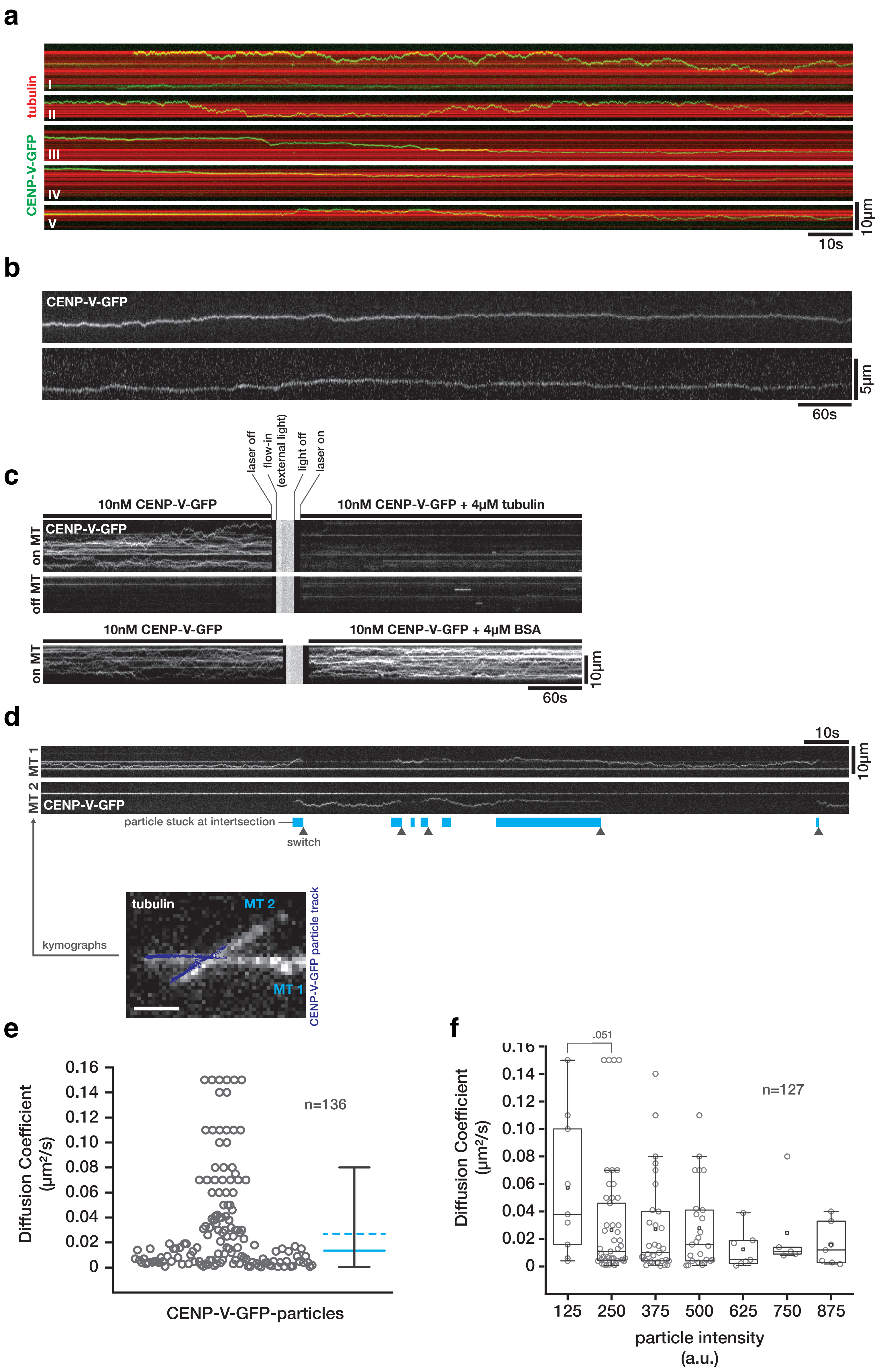

Supplemental Figure 8: Diffusional behaviour of CENP-V-GFP. (**a**) Essentially the same kymographs as shown in Fig. 5C, now showing the CENP-V-GFP signal (*green*) on top of the tubulin signal (*red*, derived from a still image of the tubulin channel that had been taken before each CENP-V-GFP movie, see Material and Methods) (**b**) Diffusion of CENP-V-GFP along the microtubule lattice. In contrast to Fig. 5C now showing kymographs of a 15-minute time-lapse movie at 1 frame per second time-resolution. (**c**) Forced MT-dissociation of CENP-V-GFP triggered by an excess of free unlabelled tubulin. Kymographs of a 10-minute time-lapse at 2 frames per second time-resolution showing the diffusion of 10 nM CENP-V-GFP preloaded onto taxol-stabilised microtubules (*first half* of the kymograph). *Vertical bright lines* in the kymographs indicate the flow-in of 10 nM CENP-V-GFP together with 4 µM free unlabelled tubulin (*upper panels*) or BSA (molecular crowding control, *lower panel*). Please note that the *horizontal lines* in the second half of the upper kymograph (*on MT*) are likely to be immobile, surface bound CENP-V-GFP particles as they are also present in a kymograph of a random line-scan off the microtubule (*off MT*). (**d**) Track-switching of a single CENP-V-eGFP particle at a microtubule intersections. *Top panels* are kymographs derived by line-scans along the intersecting substrate microtubules (MT1, MT2), shown in the bottom panel. *Grey arrowheads* indicate switch events. *Bottom panel* shows the geometry of intersecting taxol-stabilized Atto647N-labeled microtubules superimposed by the trajectory (*blue*) of the CENP-V-eGFP particle tracked by FIESTA [45]. *Please note* that superimposed data is not corrected for colour shift between channels. *Bar* equals 2 µm. (**e**) Diffusion coefficients of single CENP-V-eGFP particles derived from MSD analyses. *Solid cyan horizontal* line indicates median, *dotted line* indicates mean of data. *Error bars* indicate 1.5 IQR. (**f**) Box plot showing the diffusion coefficient of single particles (grey circles) binned by their GFP-fluorescence intensity (125 arbitrary unit bins). *Lower and upper box boundaries* indicate 25, 75 percentiles, *line* indicates median, *square* marks the mean. *Whiskers* indicate 1.5 IQR. *P-values* are derived from a two-sided Mann-Whitney U test.

Supplemental Figure 9, Nabi et al

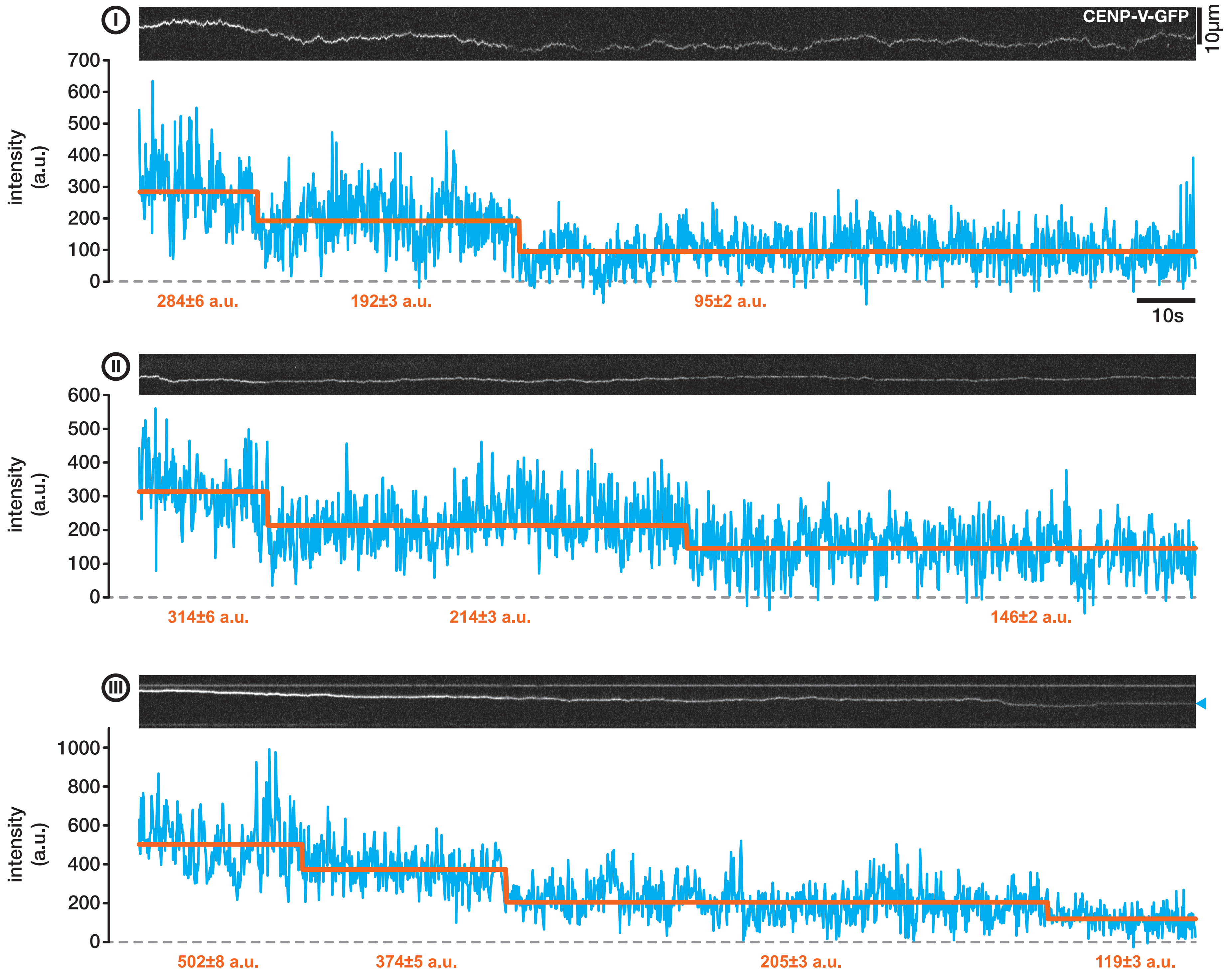

Supplemental Figure 9: Bleaching profiles of CENP-V-eGFP particles. *Top panels* are Kymographs showing the bleaching of motile CENP-V-eGFP particles. *Bottom panels* show the intensity profiles (*blue lines*) of the respective particles derived from a line-scan along the particle trace in the *kymograph above using* ImageJ**.** *Vertical red lines* indicate (manually determined) bleaching steps, *horizontal lines* the median intensity of the indicated sections in the graph. Median intensity values in arbitrary units (a.u) ± s.e.m. are given for the respective sections underneath the graph.

Supplemental Figure 10, Nabi et al

a

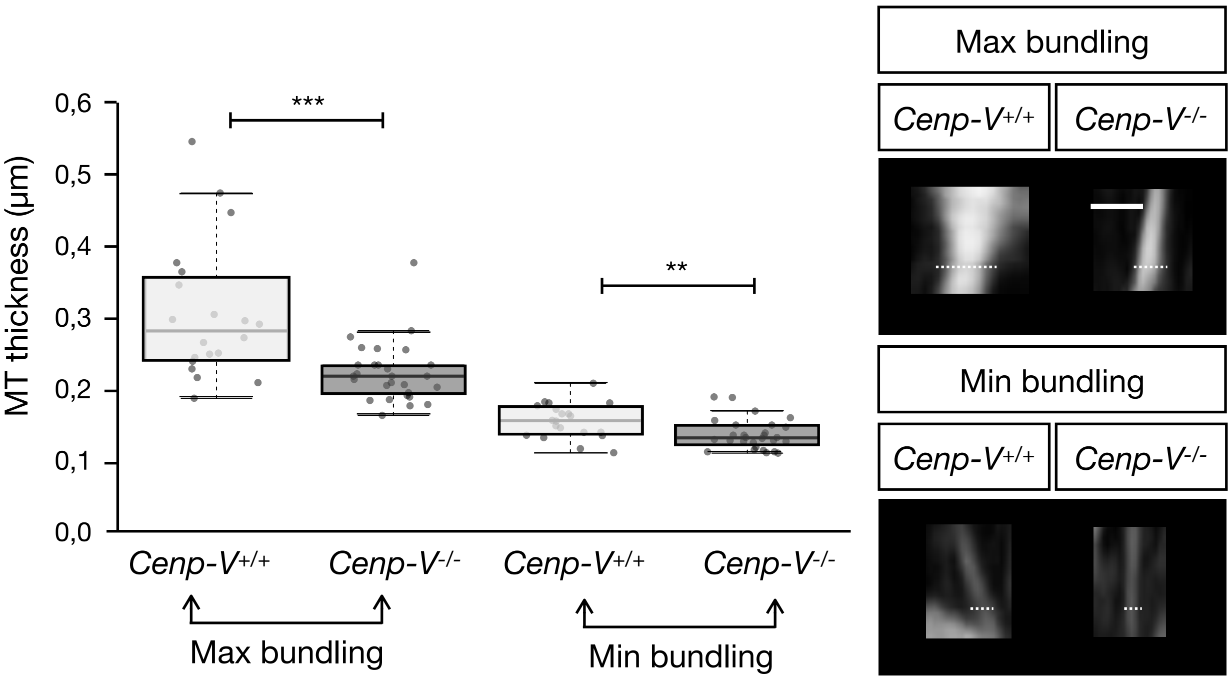

b

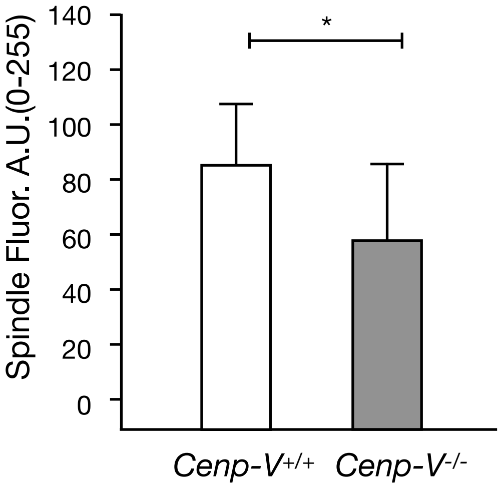

Supplemental Figure 10: Quantification of microtubule bundling in fixed *Cenp-V*$^{+/+}$ and *Cenp-V*$^{-/-}$ oocytes at MI. (a) Quantification of microtubule thickness of the most and the least prominent microtubule (maximum and minimum grade of bundling, respectively) of the meiotic spindle visualised by anti Tubulin-GFP. Scale bar= 2µm; dashed white line shows an example of the measured region. Data are represented as box plots where the middle line is the median, the lower and upper boxes correspond to the first and third quartiles and outlying points are plotted beyond the end of the error bar (1.5 IQR) n= 50 MT fibers from 3 independent experiments. **(b)** Spindle fluorescence quantification in arbitrary units (A.U.). Data were expressed as mean ± SD; n= 23 cells from 3 different experiments. (a,b) Statistical differences were tested by a paired two tailed T- test (***$p < 0.001$, **$p < 0.01$, *$p < 0.05$); the exact p values are available in the source data file. Outlier values in (a) were not included in the T-test.

Supplemental Figure 11, Nabi et al

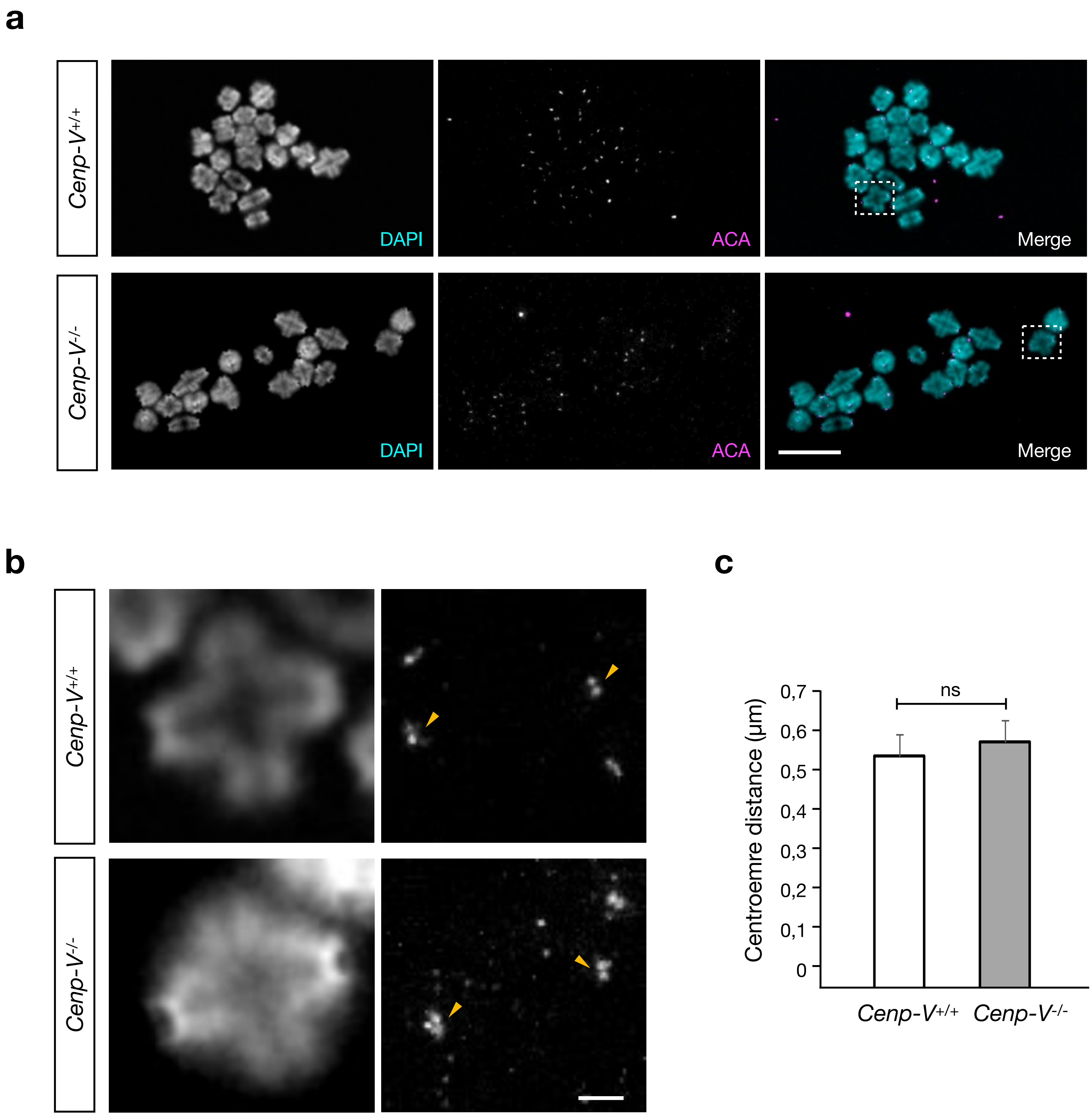

Supplemental Figure 11: Chromosome structure and centromere distance at MI. (a) Chromosome spreads at metaphase I of *Cenp-V*$^{+/+}$ and *Cenp-V*$^{-/-}$ oocytes stained with DAPI (DNA) and the centromere marker anti-ACA. Note that both genetic background show compacted chromosomes and well organised centromeres. Scale bar = 10µm. **(b)** Enlarged images of the chromosomes depicted by the dashed square in (a). The two pairs of centromeres are pointed by the yellow arrow heads, note that also neighbour centromeres are visible. Scale bar = 1µm. **(c)** Average centromere distance per cell from the experiment shown in (a,b). Data were expressed as mean ± SD; n= 100 chromosome from 3 different experiments. Statistical differences were tested by a paired two tailed T- test (ns: not significant); the exact p values are available in the source data file.
