# Supplementary material for: CENP-V is required for proper chromosome segregation through interaction with spindle microtubules in mouse oocytes
Source: Nat Commun. 2021 Nov 11;12:6547. doi: 10.1038/s41467-021-26826-3 (PMC8586017; doi:10.1038/s41467-021-26826-3)
Supplement: Supplementary file 2 — Description of Additional Supplementary Files [file 41467_2021_26826_MOESM2_ESM.pdf]

Supplemental Movie 1: Time-lapse analysis shows the progression through the first meiotic division in Cenp-$V^{+/+}$ oocytes expressing CENP-V-GFP (green) and H2B-RFP (cyan) after mRNA injection; TUBULIN is stained with SiR Tub (magenta). The movie starts just before germinal vesicle breakdown occurs. In prometaphase I, the spindle is assembled and most chromosomes are aligned in the center of the oocyte. Then the spindle migrates to the cell cortex where it expels half its chromosomal contents into a polar body (last two frames). Image stacks were acquired by Airyscan confocal every 30 minutes and a maximum intensity projection is shown for each time point. Time is shown in h:min. Movie is shown at 2 fps; n=10 cells from 2 different experiments.

Supplemental Movie 2: Time-lapse of a control oocyte expressing Tubulin-GFP (magenta) and H2B-RFP (cyan) after mRNA injection. The movie starts at 4 hours from GV stage. At 09:30 min, in metaphase I, chromosomes are perfectly aligned in the equatorial region and polar body is extruded at 11:30 min. Image stacks were acquired by SP5 confocal microscope every 30 minutes and a maximum intensity projection is shown for each time point. Time is shown in hr:min. Movie is shown at 2 fps. Scale bar =10 µm.

Supplemental Movie 3: Time-lapse of a *Cenp-*$V^{-/-}$ oocyte expressing Tubulin-GFP (magenta) and H2B-RFP (cyan) after mRNA injection. The movie starts at 4 hours from GV stage. The presence of mis-aligned chromosomes is appreciable from time 09:30 min onwards till the end which causes failure in PBE. Image stacks were acquired by SP5 confocal microscope every 30 minutes and a maximum intensity projection is shown for each time point. Time is shown in hr:min. Movie is shown at 2 fps. Scale bar =10 µm.

Supplemental Movie 4: Time-lapse of a *Cenp-*$V^{-/-}$ oocyte expressing H2B-RFP (cyan) and CENP-V GFP (green) for the rescue experiment. The movie starts at 4 hours from GV stage. The polar body is extruded at 16:00 h. Image stacks were acquired by SP5 confocal microscope every 30 minutes and a maximum intensity projection is shown for each time point. Time is shown in hr:min. Movie is shown at 2 fps. Scale bar =10 µm.

Supplemental Movie 5: Time-lapse movie showing the movement of single CENP-V-eGFP particles along taxol-stabilised ATTO 647N-labelled microtubules. CENP-V-eGFP concentrations of

1 nm (*left*) and 5 nM (*right*) are shown. The respective CENP-V-eGFP channel is shown on the *top*, an overlay of the CENP-V-eGFP channel (*cyan*) with a still from the tubulin channel (*magenta*) taken before the movie is shown on the *bottom.*

Supplemental Movie 6: Z-stack of the *Cenp-V*$^{+/+}$ metaphase I spindle shown in Fig. 6c. Planes were collected every 145 µm. Movie is shown at 2 fps. Scale bar =10 µm.

Supplemental Movie 7: Z-stack of the *Cenp-V*$^{-/-}$ metaphase I spindle shown in Fig. 6c. Planes were collected every 145 µm. Note the reduction of the k-fibers and the chromosomes misaligned in the center of the spindle compare to the *Cenp-V*$^{+/+}$ spindle shown in **Sup. Movie 6.** Movie is shown at 2 fps. Scale bar =10 µm.

Supplemental Movie 8: Z-stack of the *Cenp-V*$^{+/+}$ metaphase I spindle shown in Fig. 6f. The white arrow indicates the example of the correct two sister centromeres attachment shown in the figure. Planes were collected every 145 µm. Movie is shown at 2 fps. Scale bar =10 µm.

Supplemental Movie 9: Z-stack of the *Cenp-V*$^{-/-}$ metaphase I spindle shown in Fig. 6f. The white arrows indicate the example of sister centromeres attachments shown in the figure. In order of appearance: first arrow (00:00 sec), two sisters centromere attached; second arrow (00:08 sec), two sister centromeres unattached; third arrow (00:13 sec), only one sister centromere attached and fourth arrow (00:24 sec), two sister centromeres unattached. Note the reduction of the k-fibers in the center of the spindle and the lack of polarity compare to the *Cenp-V*$^{+/+}$ oocyte shown in **Sup. Movie 8.** Planes were collected every 145 µm. Movie is shown at 2 fps. Scale bar = 10µm.

Supplemental Movie 10: Z-stack of the *Cenp-V*$^{+/+}$ metaphase II spindle shown in Fig. 7e. The white arrow indicates the example of amphitelic attachment shown in the figure. Planes were collected every 145 µm. Movie is shown at 2fps. Scale bar = 10µm.

Supplemental Movie 11: Z-stack of the *Cenp-V*$^{-/-}$ metaphase II spindle shown in Fig. 7e. Note the reduction of the k-fibers around the chromosomes and the lack of polarity compare to the *Cenp-*

V$^{+/+}$ oocyte shown in **Sup. Movie 10.** Planes were collected every 145 µm. Movie is shown at 2 fps. Scale bar = 10µm.
