# Supplementary material for: CENP-V is required for proper chromosome segregation through interaction with spindle microtubules in mouse oocytes
Source: Nat Commun. 2021 Nov 11;12:6547. doi: 10.1038/s41467-021-26826-3 (PMC8586017; doi:10.1038/s41467-021-26826-3)
Supplement: Supplementary file 14 — Reporting Summary [file 41467_2021_26826_MOESM14_ESM.pdf]

nature portfolio

Double-blind peer review submissions: write DBPR and your manuscript number here

Corresponding author(s): RJEISSBERGER, M. CHACON

Last updated by author(s): 2021-07-12

Reporting Summary

Nature Portfolio wishes to improve the reproducibility of the work that we publish. This form provides structure for consistency and transparency in reporting. For further information on Nature Portfolio policies, see our Editorial Policies and the Editorial Policy Checklist.

Statistics

For all statistical analyses, confirm that the following items are present in the figure legend, table legend, main text, or Methods section.

n/a	Confirmed	
[ ]	[x]	The exact sample size (n) for each experimental group/condition, given as a discrete number and unit of measurement
[ ]	[x]	A statement on whether measurements were taken from distinct samples or whether the same sample was measured repeatedly
[ ]	[x]	The statistical test(s) used AND whether they are one- or two-sided<br>*Only common tests should be described solely by name; describe more complex techniques in the Methods section.*
[x]	[ ]	A description of all covariates tested
[x]	[ ]	A description of any assumptions or corrections, such as tests of normality and adjustment for multiple comparisons
[ ]	[x]	A full description of the statistical parameters including central tendency (e.g. means) or other basic estimates (e.g. regression coefficient) AND variation (e.g. standard deviation) or associated estimates of uncertainty (e.g. confidence intervals)
[ ]	[x]	For null hypothesis testing, the test statistic (e.g. F, t, r) with confidence intervals, effect sizes, degrees of freedom and P value noted<br>*Give P values as exact values whenever suitable.*
[x]	[ ]	For Bayesian analysis, information on the choice of priors and Markov chain Monte Carlo settings
[x]	[ ]	For hierarchical and complex designs, identification of the appropriate level for tests and full reporting of outcomes
[ ]	[x]	Estimates of effect sizes (e.g. Cohen's d, Pearson's r), indicating how they were calculated

Our web collection on statistics for biologists contains articles on many of the points above.

Software and code

Policy information about availability of computer code

Data collection	n/a
Data analysis	Fiji/ImageJ v 2.0.0-RC-41/1.50D; Excel v 15.13.3, GraphPadPrism, MATLAB R2018A

For manuscripts utilizing custom algorithms or software that are central to the research but not yet described in published literature, software must be made available to editors and reviewers. We strongly encourage code deposition in a community repository (e.g. GitHub). See the Nature Portfolio guidelines for submitting code & software for further information.

Data

Policy information about availability of data

All manuscripts must include a data availability statement. This statement should provide the following information, where applicable:

- Accession codes, unique identifiers, or web links for publicly available datasets
- A description of any restrictions on data availability
- For clinical datasets or third party data, please ensure that the statement adheres to our policy

Provide your data availability statement here.

Field-specific reporting

Please select the one below that is the best fit for your research. If you are not sure, read the appropriate sections before making your selection.

☒ Life sciences ☐ Behavioural & social sciences ☐ Ecological, evolutionary & environmental sciences

For a reference copy of the document with all sections, see nature.com/documents/nr-reporting-summary-flat.pdf

Life sciences study design

All studies must disclose on these points even when the disclosure is negative.

Sample size	The sample size is different for each type of experiment, but for all experiments at least 3 female mice of each, control and "knockout" were used, often many more (> 10). At least 10, often many more oocytes of each mouse were analyzed. Specifics are provided in the figure legends. These sample sizes were considered fully sufficient as the phenotypes were easily and constantly observed.
Data exclusions	No data were excluded
Replication	Experiments were repeated at least 3 times, often many more times and except for obvious technical failures, the results were consistently reproduced.
Randomization	There were only 2 groups: control and "knockout" oocytes, thus no randomization was applied.
Blinding	Blinding was not possible as the "knockout" oocytes were easy to identify. No other experiment required blinding.

Reporting for specific materials, systems and methods

We require information from authors about some types of materials, experimental systems and methods used in many studies. Here, indicate whether each material, system or method listed is relevant to your study. If you are not sure if a list item applies to your research, read the appropriate section before selecting a response.

Materials & experimental systems

n/a	Involved in the study
☐	☒ Antibodies
☐	☒ Eukaryotic cell lines
☒	☐ Palaeontology and archaeology
☐	☒ Animals and other organisms
☒	☐ Human research participants
☒	☐ Clinical data
☒	☐ Dual use research of concern

Methods

n/a	Involved in the study
☒	☐ ChIP-seq
☒	☐ Flow cytometry
☒	☐ MRI-based neuroimaging

Antibodies

Antibodies used	Primary antibodies used were as follows: rabbit anti-CENP-V (HPA042616; Sigma) at 1:100, rabbit anti-CENP-V (homemade) at 1:250, rat anti-α-tubulin (MCA78G; Bio-Rad) at 1:5000, mouse anti-SYCP3 (clone 60C10) a gift from Dr. C. Heyting at 1:500, mouse anti-γ-tubulin (ab11316; Abcam) at 1:5000, human anti-ACA (ANI-15-235-000; Biozol) at 1:50, goat anti-CENP-A (sc-11277; Santa Cruz) at 1:100. Secondary antibodies were used as follow: anti-rabbit Alexa Fluor 488 (A27034; Invitrogen) at 1:500, anti-mouse Alexa Fluor 647 (715-605-151; Jackson Immuno Research) at 1:500, anti-mouse Alexa Fluor 568 (715-605-151; Invitrogen) at 1:500, anti-human Alexa Fluor 568 (A-21090; Invitrogen) at 1:500, anti-sheep Alexa Fluor 568 (A-21099; Invitrogen) at 1:500, anti-goat Alexa Fluor 488 (ab150129; abcam).
Validation	The self-made rabbit anti-CENP-V was validated and titrated by/in immunofluorescence and Western blot approaches in wild-type and CENP-V deficient cells. There was no signal in CENP-V deficient cells. Anti-α-tubulin (MCA78G; Bio-Rad) antibody has been validated by Bio-Rad by demonstrating immunoblotting and immunostainings on different cell lines (see website). Anti-γ-tubulin (ab11316; Abcam) antibody has been validated by abcam through immunocytochemistry and western blot techniques (see website). Anti-ACA (ANI-15-235-0001; Biozol) specificity was validated by immunofluorescence on spermatocytes and oocytes chromosomes spreads together with other centromeric markers as anti-CENP-A. Anti-SYCP3 (clone 60C10) antibody was validated in our and many other previous publications (e.g. Biswas et al, 2018). Anti-CENP-A (sc-11277; Santa Cruz) has been validated for use in immunohistochemistry and western blotting, as stated on the Santa Cruz product page.

Eukaryotic cell lines

Policy information about cell lines

Cell line source(s)	Internal stock
Authentication	SF9 cell line, used only to produce recombinant protein
Mycoplasma contamination	tested negative

Commonly misidentified lines
(See ICLAC register)

Name any commonly misidentified cell lines used in the study and provide a rationale for their use.

Palaeontology and Archaeology

Specimen provenance

Provide provenance information for specimens and describe permits that were obtained for the work (including the name of the issuing authority, the date of issue, and any identifying information). Permits should encompass collection and, where applicable, export.

Specimen deposition

Indicate where the specimens have been deposited to permit free access by other researchers.

Dating methods

If new dates are provided, describe how they were obtained (e.g. collection, storage, sample pretreatment and measurement), where they were obtained (i.e. lab name), the calibration program and the protocol for quality assurance OR state that no new dates are provided.

☐ Tick this box to confirm that the raw and calibrated dates are available in the paper or in Supplementary Information.

Ethics oversight

Identify the organization(s) that approved or provided guidance on the study protocol, OR state that no ethical approval or guidance was required and explain why not.

Note that full information on the approval of the study protocol must also be provided in the manuscript.

Animals and other organisms

Policy information about studies involving animals; ARRIVE guidelines recommended for reporting animal research

Laboratory animals

To generate a germ cell specific CENP-V deficiency, we have obtained three clones of ES cells in which Exon 3 of the gene is flanked by loxP sites to be recombined by Cre recombinase. An insertion cassette has to be deleted first by using FLP recombinase, which recombines the FRT sites. We have obtained highly (90 %) chimeric mice and have bred them with C57BL/6 mice. Germline transmission was obtained and Cenp-Vfl/+ mice were bred to the Flpo transgenic strain expressing FLP recombinase. Cenp-Vfl/+neo- progeny was obtained and breaded with germ cell specific Cre-expressing mouse strains. We used the following two strains, all of which have been shown to efficiently delete target genes in germ cells: (1) Vasa-Cre, which expresses Cre in late spermatogonia in males and at entry into meiosis in females; (2) GDF9-Cre, which expresses Cre in females at day 2 after birth, and (3) SPO11-Cre, where Cre is expressed in male and female early prophase.

To generate a germ cell specific CENP-V deficiency, we have obtained three clones of ES cells in which Exon 3 of the gene is flanked by loxP sites to be recombined by Cre recombinase. An insertion cassette has to be deleted first by using FLP recombinase, which recombines the FRT sites. We have obtained highly (90 %) chimeric mice and have bred them with C57BL/6 mice. Germline transmission was obtained and Cenp-Vfl/+ mice were bred to the Flpo transgenic strain expressing FLP recombinase. Cenp-Vfl/+neo- progeny was obtained and breaded with germ cell specific Cre-expressing mouse strains. We used the following two strains, all of which have been shown to efficiently delete target genes in germ cells: (1) Vasa-Cre, which expresses Cre in late

Wild animals

No wild animals were used

[illegible]erved in or captured in the field; report species, sex and age where possible. Describe how animals were caught and transported and what happened to captive animals after the study (if killed, explain why and describe method; if released, say where and when) OR state that the study did not involve wild animals.

Field-collected samples

No field-collected samples were used

[illegible]ples, describe all relevant parameters such as housing, maintenance, temperature, photoperiod and end-of-experiment protocol OR state that the study did not involve samples collected from the field.

Ethics oversight

All mice were bred and maintained under a 12 hr light/12 hr dark cycle in the animal facility of the medical faculty, Technische Universität Dresden, according to approved institutional guidelines. All experiments were approved by the state animal welfare office.

Note that full information on the approval of the study protocol must also be provided in the manuscript.

Human research participants

Policy information about studies involving human research participants

Population characteristics

Describe the covariate-relevant population characteristics of the human research participants (e.g. age, gender, genotypic information, past and current diagnosis and treatment categories). If you filled out the behavioural & social sciences study design questions and have nothing to add here, write "See above."

Recruitment

Describe how participants were recruited. Outline any potential self-selection bias or other biases that may be present and how these are likely to impact results.

Ethics oversight

Identify the organization(s) that approved the study protocol.

Note that full information on the approval of the study protocol must also be provided in the manuscript.

Clinical data

Policy information about clinical studies

All manuscripts should comply with the ICMJE guidelines for publication of clinical research and a completed CONSORT checklist must be included with all submissions.

Clinical trial registration	*Provide the trial registration number from ClinicalTrials.gov or an equivalent agency.*
Study protocol	*Note where the full trial protocol can be accessed OR if not available, explain why.*
Data collection	*Describe the settings and locales of data collection, noting the time periods of recruitment and data collection.*
Outcomes	*Describe how you pre-defined primary and secondary outcome measures and how you assessed these measures.*

Dual use research of concern

Policy information about dual use research of concern

Hazards

Could the accidental, deliberate or reckless misuse of agents or technologies generated in the work, or the application of information presented in the manuscript, pose a threat to:

No	Yes	
☒	☐	Public health
☒	☐	National security
☒	☐	Crops and/or livestock
☒	☐	Ecosystems
☒	☐	Any other significant area

Experiments of concern

Does the work involve any of these experiments of concern:

No	Yes	
☒	☐	Demonstrate how to render a vaccine ineffective
☒	☐	Confer resistance to therapeutically useful antibiotics or antiviral agents
☒	☐	Enhance the virulence of a pathogen or render a nonpathogen virulent
☒	☐	Increase transmissibility of a pathogen
☒	☐	Alter the host range of a pathogen
☒	☐	Enable evasion of diagnostic/detection modalities
☒	☐	Enable the weaponization of a biological agent or toxin
☒	☐	Any other potentially harmful combination of experiments and agents

ChIP-seq

Data deposition

☐ Confirm that both raw and final processed data have been deposited in a public database such as GEO.

☐ Confirm that you have deposited or provided access to graph files (e.g. BED files) for the called peaks.

Data access links<br>*May remain private before publication.*	*For "Initial submission" or "Revised version" documents, provide reviewer access links. For your "Final submission" document, provide a link to the deposited data.*
Files in database submission	*Provide a list of all files available in the database submission.*
Genome browser session<br>(e.g. UCSC)	*Provide a link to an anonymized genome browser session for "Initial submission" and "Revised version" documents only, to enable peer review. Write "no longer applicable" for "Final submission" documents.*

Methodology

Replicates	*Describe the experimental replicates, specifying number, type and replicate agreement.*
Sequencing depth	*Describe the sequencing depth for each experiment, providing the total number of reads, uniquely mapped reads, length of reads and*

Sequencing depth

whether they were paired- or single-end.

Antibodies

Describe the antibodies used for the ChIP-seq experiments; as applicable, provide supplier name, catalog number, clone name, and lot number.

Peak calling parameters

Specify the command line program and parameters used for read mapping and peak calling, including the ChIP, control and index files used.

Data quality

Describe the methods used to ensure data quality in full detail, including how many peaks are at FDR 5% and above 5-fold enrichment.

Software

Describe the software used to collect and analyze the ChIP-seq data. For custom code that has been deposited into a community repository, provide accession details.

Flow Cytometry

Plots

Confirm that:

- [ ] The axis labels state the marker and fluorochrome used (e.g. CD4-FITC).
- [ ] The axis scales are clearly visible. Include numbers along axes only for bottom left plot of group (a 'group' is an analysis of identical markers).
- [ ] *All plots are contour plots with outliers or pseudocolor plots.*
- [ ] A numerical value for number of cells or percentage (with statistics) is provided.

Methodology

Sample preparation

Describe the sample preparation, detailing the biological source of the cells and any tissue processing steps used.

Instrument

Identify the instrument used for data collection, specifying make and model number.

Software

Describe the software used to collect and analyze the flow cytometry data. For custom code that has been deposited into a community repository, provide accession details.

Cell population abundance

Describe the abundance of the relevant cell populations within post-sort fractions, providing details on the purity of the samples and how it was determined.

Gating strategy

Describe the gating strategy used for all relevant experiments, specifying the preliminary FSC/SSC gates of the starting cell population, indicating where boundaries between "positive" and "negative" staining cell populations are defined.

- [ ] Tick this box to confirm that a figure exemplifying the gating strategy is provided in the Supplementary Information.

Magnetic resonance imaging

Experimental design

Design type

Indicate task or resting state; event-related or block design.

Design specifications

Specify the number of blocks, trials or experimental units per session and/or subject, and specify the length of each trial or block (if trials are blocked) and interval between trials.

Behavioral performance measures

State number and/or type of variables recorded (e.g. correct button press, response time) and what statistics were used to establish that the subjects were performing the task as expected (e.g. mean, range, and/or standard deviation across subjects).

Acquisition

Imaging type(s)

Specify: functional, structural, diffusion, perfusion.

Field strength

Specify in Tesla

Sequence & imaging parameters

Specify the pulse sequence type (gradient echo, spin echo, etc.), imaging type (EPI, spiral, etc.), field of view, matrix size, slice thickness, orientation and TE/TR/flip angle.

Area of acquisition

State whether a whole brain scan was used OR define the area of acquisition, describing how the region was determined.

Diffusion MRI ☐ Used ☐ Not used

Preprocessing

Preprocessing software	Provide detail on software version and revision number and on specific parameters (model/functions, brain extraction, segmentation, smoothing kernel size, etc.).
Normalization	If data were normalized/standardized, describe the approach(es): specify linear or non-linear and define image types used for transformation OR indicate that data were not normalized and explain rationale for lack of normalization.
Normalization template	Describe the template used for normalization/transformation, specifying subject space or group standardized space (e.g. original Talairach, MNI305, ICBM152) OR indicate that the data were not normalized.
Noise and artifact removal	Describe your procedure(s) for artifact and structured noise removal, specifying motion parameters, tissue signals and physiological signals (heart rate, respiration).
Volume censoring	Define your software and/or method and criteria for volume censoring, and state the extent of such censoring.

Statistical modeling & inference

Model type and settings	Specify type (mass univariate, multivariate, RSA, predictive, etc.) and describe essential details of the model at the first and second levels (e.g. fixed, random or mixed effects; drift or auto-correlation).
Effect(s) tested	Define precise effect in terms of the task or stimulus conditions instead of psychological concepts and indicate whether ANOVA or factorial designs were used.

Specify type of analysis: ☐ Whole brain ☐ ROI-based ☐ Both

Statistic type for inference (See Eklund et al. 2016)	Specify voxel-wise or cluster-wise and report all relevant parameters for cluster-wise methods.
Correction	Describe the type of correction and how it is obtained for multiple comparisons (e.g. FWE, FDR, permutation or Monte Carlo).

Models & analysis

n/a	Involved in the study
☐	☐ Functional and/or effective connectivity
☐	☐ Graph analysis
☐	☐ Multivariate modeling or predictive analysis

Functional and/or effective connectivity	Report the measures of dependence used and the model details (e.g. Pearson correlation, partial correlation, mutual information).
Graph analysis	Report the dependent variable and connectivity measure, specifying weighted graph or binarized graph, subject- or group-level, and the global and/or node summaries used (e.g. clustering coefficient, efficiency, etc.).
Multivariate modeling and predictive analysis	Specify independent variables, features extraction and dimension reduction, model, training and evaluation metrics.

nature portfolio

Corresponding author(s): Rolf Jessberger, Mariola Chacon

Last updated by author(s): 2021-7-12

Editorial Policy Checklist

This form is used to ensure compliance with Nature Portfolio editorial policies related to research ethics and reproducibility. For further information, please see our editorial policies site. All relevant questions on the form must be answered.

Competing interests

Policy information about competing interests

Competing interests declaration

In the interest of transparency and to help readers form their own judgements of potential bias, Nature Portfolio journals require authors to declare any competing financial and/or non-financial interest in relation to the work described in the submitted manuscript.

☒ We declare that none of the authors have competing financial or non-financial interests as defined by Nature Portfolio.

☐ We declare that one or more of the authors have a competing interest as defined by Nature Portfolio.

Authorship

Policy information about authorship

Prior to submission all listed authors must agree to all manuscript contents, the author list and its order and the author contribution statements. Any changes to the author list after submission must be approved by all authors.

☒ We have read the Nature Portfolio Authorship Policy and confirm that this manuscript complies.

Data availability

Policy information about availability of data

Data availability statement

All manuscripts must include a data availability statement. This statement should provide the following information, where applicable:

- Accession codes, unique identifiers, or web links for publicly available datasets
- A description of any restrictions on data availability
- *For clinical datasets or third party data, please ensure that the statement adheres to our policy*

☐ We have provided a full data availability statement in the manuscript.

Mandated accession codes (where applicable)

Confirm that all relevant data are deposited into a public repository and that accession codes are provided.

☐ All relevant accession codes are provided ☐ Accession codes will be available before publication ☒ No data with mandated deposition

Code availability

Policy information about availability of computer code

Code availability statement

For all studies using custom code or mathematical algorithm that is deemed central to the conclusions, the manuscript must include a statement under the heading "Code availability" describing how readers can access the code, including any access restrictions. Code availability statements should be provided as a separate section after the data availability statement but before the References.

☐ We have provided a full code availability statement in the manuscript

Data presentation

For all data presented in a plot, chart or other visual representation confirm that:

n/a	Confirmed	
☐	☒	Individual data points are shown when possible, and always for n ≤ 10
☐	☒	The format shows data distribution clearly (e.g. dot plots, box-and-whisker plots)
☐	☒	Box-plot elements are defined (e.g. center line, median; box limits, upper and lower quartiles; whiskers, 1.5x interquartile range; points, outliers)
☐	☒	Clearly defined error bars are present and what they represent (SD, SE, CI) is noted

Image integrity

Policy information about image integrity

☒ We have read Nature Portfolio's image integrity policy and all images comply.

Unprocessed data must be provided upon request. Please double-check figure assembly to ensure that all panels are accurate (e.g. all labels are correct, no inadvertent duplications have occurred during preparation, etc.).

Where blots and gels are presented, please take particular care to ensure that lanes have not been spliced together, that loading controls are run on the same blot, and that unprocessed scans match the corresponding figures.

Additional policy considerations

Some types of research require additional policy disclosures. Please indicate whether each of these apply to your study. If you are not certain, please read the appropriate section before selecting a response.

Does not apply	Involved in the study	
☒	☐	Macromolecular structural data
☐	☒	Unique biological materials
☐	☒	Research animals and/or animal-derived materials that require ethical approval
☒	☐	Human embryos, gametes and/or stem cells
☒	☐	Human research participants
☒	☐	Clinical data
☒	☐	Archaeological, geological, and palaeontological materials

Biological materials

Policy information about availability of materials

Obtaining biological materials: *We have generated the CENP-V deficient mouse strain and will make it available to the scientific community for research purposes.*

☐ We have described these restrictions in the manuscript. ☐ We have described how to obtain all materials in the manuscript.

Research animals

Policy information about studies involving animals; ARRIVE guidelines recommended for reporting animal research

Ethical compliance

☒ We have complied with all relevant ethical regulations and include a statement affirming this in the manuscript.

Ethics committee

☒ We have disclosed the name(s) of the board and institution that approved the study protocol in the manuscript.

I certify that all the above information is complete and correct.

Typed signature: Rolf Jessberger   Date: 2021-7-12

This checklist template is licensed under a Creative Commons Attribution 4.0 International License, which permits use, sharing, adaptation, distribution and reproduction in any medium or format, as long as you give appropriate credit to the original author(s) and the source, provide a link to the Creative Commons license, and indicate if changes were made. The images or other third party material in this article are included in the article's Creative Commons license, unless indicated otherwise in a credit line to the material. If material is not included in the article's Creative Commons license and your intended use is not permitted by statutory regulation or exceeds the permitted use, you will need to obtain permission directly from the copyright holder. To view a copy of this license, visit http://creativecommons.org/licenses/by/4.0/
